# De Novo Mutation and Rapid Protein (Co-)evolution during Meiotic Adaptation in *Arabidopsis arenosa*

**DOI:** 10.1093/molbev/msab001

**Published:** 2021-01-27

**Authors:** Magdalena Bohutínská, Vinzenz Handrick, Levi Yant, Roswitha Schmickl, Filip Kolář, Kirsten Bomblies, Pirita Paajanen

**Affiliations:** 1 Department of Botany, Faculty of Science, Charles University, Prague, Czech Republic; 2 Institute of Botany of the Czech Academy of Sciences, Průhonice, Czech Republic; 3 Department of Cell and Developmental Biology, John Innes Centre, Norwich, United Kingdom; 4 Department of Botany, University of Innsbruck, Innsbruck, Austria; 5 Plant Evolutionary Genetics, Department of Biology, Institute of Molecular Plant Biology, ETH Zürich, Zurich, Switzerland

**Keywords:** de novo mutations, standing variation, coevolution, meiosis, polyploidy

## Abstract

A sudden shift in environment or cellular context necessitates rapid adaptation. A dramatic example is genome duplication, which leads to polyploidy. In such situations, the waiting time for new mutations might be prohibitive; theoretical and empirical studies suggest that rapid adaptation will largely rely on standing variation already present in source populations. Here, we investigate the evolution of meiosis proteins in *Arabidopsis arenosa*, some of which were previously implicated in adaptation to polyploidy, and in a diploid, habitat. A striking and unexplained feature of prior results was the large number of amino acid changes in multiple interacting proteins, especially in the relatively young tetraploid. Here, we investigate whether selection on meiosis genes is found in other lineages, how the polyploid may have accumulated so many differences, and whether derived variants were selected from standing variation. We use a range-wide sample of 145 resequenced genomes of diploid and tetraploid *A. arenosa*, with new genome assemblies. We confirmed signals of positive selection in the polyploid and diploid lineages they were previously reported in and find additional meiosis genes with evidence of selection. We show that the polyploid lineage stands out both qualitatively and quantitatively. Compared with diploids, meiosis proteins in the polyploid have more amino acid changes and a higher proportion affecting more strongly conserved sites. We find evidence that in tetraploids, positive selection may have commonly acted on de novo mutations. Several tests provide hints that coevolution, and in some cases, multinucleotide mutations, might contribute to rapid accumulation of changes in meiotic proteins.

## Introduction

Sometimes an abrupt change in circumstances forces a rapid evolutionary response. As populations face new challenges, positive selection can act on alleles recruited from standing variation or on de novo mutations (Barrett and Schluter 2008). Though in long-term macroevolution, de novo mutations clearly play a role, evolution from standing variation may be especially important in facilitating rapid adaptation, because it eliminates the waiting time needed for novel mutations (Hermisson and Pennings 2005; Prezeworski et al. 2005; Barrett and Schluter 2008). There are numerous reports of rapid adaptation to novel environments that utilize standing genetic variation (Jones et al. 2012; Van Belleghem et al. 2018; Haenel et al. 2019; Lai et al. 2019), whereas reports of de novo mutations in such instances are rare and often include loss of function mutations (Messer and Petrov 2013; Exposito-Alonso et al. 2018; Wu et al. 2018; Xie et al. 2019). However, it is also predicted that de novo variants may have stronger phenotypic effects than standing variants (Matuszewski et al. 2015). Thus, the relative importance of de novo mutations may be greater when extensive functional restructuring is needed.

**Fig. 1.**
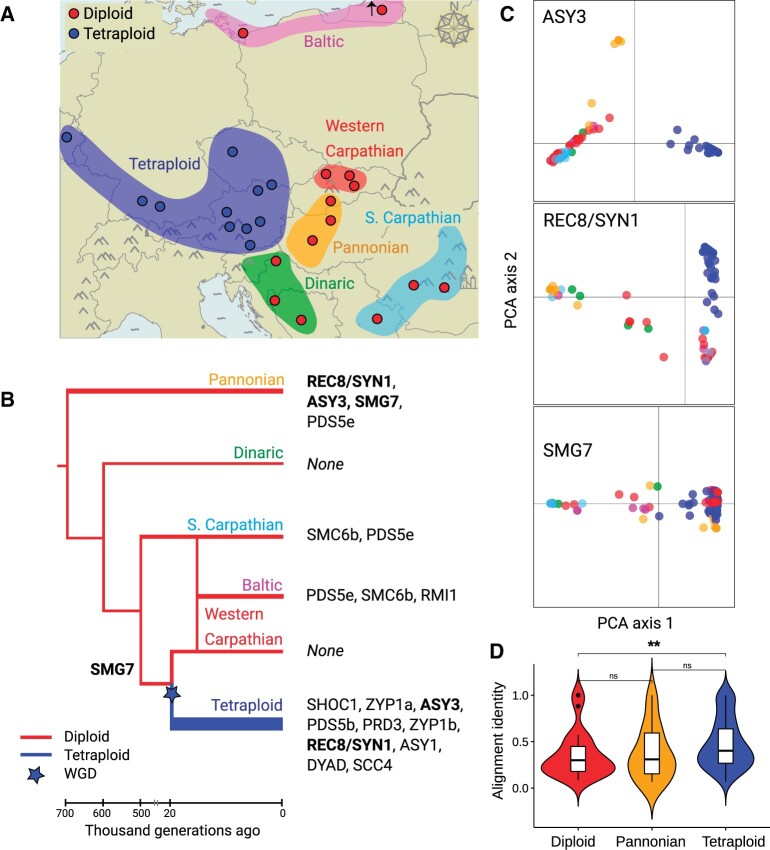
Meiosis proteins showing signatures of positive selection in *Arabidopsis arenosa* lineages. (*A*) Our sampling of *A. arenosa* populations in Europe. Dots show 14 diploid (red) and 11 tetraploid populations without signs of introgression from diploids (blue) studied here. Distribution ranges of all known *A. arenosa* lineages are shown as colored areas, indicating that our sampling covers a complete diversity of diploid lineages (based on Kolář et al. 2016; Monnahan et al. 2019). The tetraploid distribution range covers areas occupied by populations without signs of introgression from diploids (Monnahan et al. 2019). (*B*) Phylogeny of *A. arenosa* (based on Kolář et al. 2016; Monnahan et al. 2019) with candidate meiosis proteins placed on the branch where they exhibit signatures of selective sweeps (identified as *F*_ST_ and FineMAV overlap, see the main text). Width of the branches corresponds to the number of meiosis proteins that are identified as positive selection candidates. Only *Pannonian* and tetraploid lineages had more meiosis proteins showing signatures of positive selection than expected by chance. Lineages with no evidence for positive selection on meiosis proteins are indicated as “None.” Proteins are ordered from those having the highest number of candidate AASs to the lowest (supplementary tables S5 and S6, Supplementary Material online). Three proteins found independently as candidates in parallel in two lineages are written in bold. Time axis below the tree indicates median estimates of lineage divergence times (based on Arnold et al. 2015; Kolář et al. 2016). (*C*) Principal component analysis based on allele frequencies of candidate AASs in the three parallel candidate meiosis proteins. Each dot represents one individual, colored based on lineage in panel A. (*D*) positive selection targeted more conserved amino acids in tetraploids (blue) than in diploids (red; summarizing candidate AASs identified in all but the *Pannonian* diploid lineage—orange). Each violin plot summarizes alignment identity (calculated across 17 plant reference genomes, higher value indicate more conserved site) over all candidate AASs identified in the corresponding lineage. **P = 0.002, Wilcoxon rank sum test.

Whole-genome duplication, which leads to polyploidy, is an example of a situation where the cellular context suddenly and substantially shifts, necessitating a rapid adaptive response (Comai 2005; Bomblies and Madlung 2014). Previous studies on the genetic basis of adaptation to genome duplication in the diploid–autotetraploid species, A*rabidopsis arenosa* (fig. 1*A*), identified a set of genes showing strong evidence of positive selection in its tetraploid lineage (Hollister et al. 2012; Yant et al. 2013; Wright et al. 2015). Many of these genes encode interacting proteins important for meiosis, which is consistent with the fact that meiosis is particularly challenged by genome duplication (Comai 2005; Cifuentes et al. 2010; Stenberg and Saura 2013; Bomblies and Madlung 2014; Bomblies et al. 2016). That at least some of these changes are adaptive is supported by the observation that the derived alleles of two of the genes, which encode interacting meiotic axis proteins, have been experimentally shown to affect meiotic traits relevant to tetraploid meiotic stability (Morgan et al. 2020).

Meiosis is a structurally conserved process that is periodically challenged and driven to evolve in diploids as well (Heyting 1996; Kumar et al. 2010; Grishaeva and Bogdanov 2014; Bomblies et al. 2015; Baker et al. 2017; Brand et al. 2019). But what was striking in the *A. arenosa* tetraploids, and remains unexplained, is that although two independent estimates suggest the tetraploids are likely only about 20,000–30,000 generations old (fig. 1*B*; Arnold et al. 2015; Monnahan et al. 2019), a surprisingly large number of amino acid changes differentiates ancestral diploid and derived tetraploid alleles in the subset of meiosis genes that have signatures of positive selection. Meanwhile, the rest of the genome, including other meiosis genes, remains largely undifferentiated (Hollister et al. 2012, Yant et al. 2013). Another study showed that positive selection on meiosis is not unique to the tetraploid *A. arenosa* lineage: Signatures of selection were also found in two of the same meiosis genes (different alleles) in a distinct diploid *A. arenosa* lineage (Wright et al. 2015). This raised the possibility that rapid evolution of meiosis genes might be a common feature of *A. arenosa* lineages regardless of ploidy, and this is one of the ideas we test here.

The above observations leave many questions about the evolution of meiosis in *A. arenosa* lineages unanswered, which also have wider implications for understanding rapid evolutionary adaptation of essential cellular processes. Remaining questions include: Is the evolution of meiosis in the tetraploid lineage more likely to have targeted functionally important sites than in diploids? Were the variants that selection acted on in the tetraploid lineage already present as standing variation in diploids? If not, what might drive the rapid accumulation of multiple amino acid changes in these proteins? To address such questions, we analyzed a range-wide data set of 145 diploid and tetraploid *A. arenosa* genome sequences (fig. 1*A*; Monnahan et al. 2019), sampling four additional diploid lineages not previously included, complemented with newly generated assemblies for the diploid and tetraploid that allowed us to define haplotypes more reliably. We found that although evidence of positive selection on meiosis proteins is not unique to the tetraploid lineage, the extent of meiotic protein remodeling is. Moreover, we found evidence that selection likely acted at least in part on de novo mutations not present in the diploid gene pool. We also find support for the idea that coevolution of proteins and the accumulation of multinucleotide mutations could contribute to the de novo accumulation of many amino acid variants in the tetraploid lineage.

## Results and Discussion

### Meiosis Protein Evolution in *A. arenosa* Lineages

We investigated the patterns of evolution of meiosis proteins across all currently known *A. arenosa* lineages (fig. 1*A*), including samples of four additional diploid lineages in which meiosis protein evolution was not investigated in our previous study (Wright et al. 2015). This additional sampling allowed us to ask whether positive selection commonly targets meiosis in different diploid and tetraploid lineages (i.e., whether selection on meiosis is the rule rather than the exception). This sampling also allowed us to investigate whether the patterns in the tetraploid lineage are qualitatively or quantitatively unusual. We did this using a published data set of single nucleotide polymorphism (SNP) variation that includes range-wide sampling of diploid and tetraploid whole-genome resequenced individuals (Monnahan et al. 2019; see supplementary table S1, Supplementary Material online), complemented with two new genome assemblies of diploid and tetraploid individuals using the 10× genomics Chromium platform and supernova assembler (Weisenfeld et al. 2017; see supplementary table S2, Supplementary Material online). These new assemblies allowed us to extract diploid- and tetraploid-specific haplotypes for candidate genes (see Materials and Methods for details). We focused on protein sequence evolution, as this allows us to capitalize on the availability of tests that can help assess which changes are likely to be functional.

We first asked whether evidence of selection on meiosis genes is unique to the two lineages, it was previously reported in (the tetraploid and Pannonian diploids; Wright et al. 2015), or is consistently seen across *A. arenosa* lineages (i.e., to ask if this is a ubiquitous feature of meiotic protein evolution). We focused on a list of 78 meiosis-specific proteins (supplementary table S3, Supplementary Material online) selected by refining available lists (Sánchez-Morán et al. 2005; Yant et al. 2013) using the Pathway Interaction Database (PID; Schaefer et al. 2009), AraNet (Lee et al. 2015), and TAIR databases (Berardini et al. 2015). We also confirmed that diploid and tetraploid populations included in our analyses had similar genetic diversity and allele frequency spectra (Monnahan et al. 2019, supplementary table S4, Supplementary Material online), indicating a lack of severe demographic change such as recent population expansions or bottlenecks that could otherwise have had a confounding effect on our analyses.

**Fig. 2.**
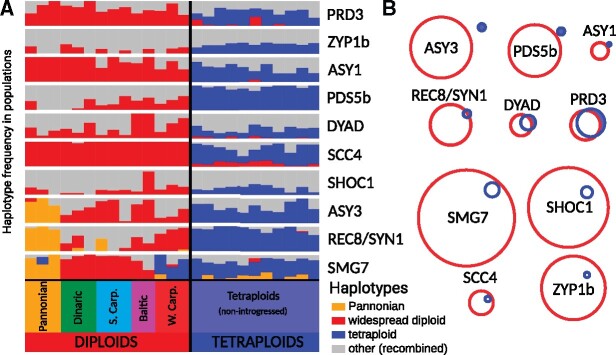
Limited standing variation across *Arabidopsis arenosa* diploids in protein candidates for tetraploid meiotic adaptation. (*A*) Lack of tetraploid-specific haplotypes in diploid populations sampled across the total range of *A. arenosa*. Haplotypes were combined across linked candidate AASs within each protein. A set of bar plots for each of ten candidate proteins (horizontal lines) shows frequencies of diploid, *Pannonian* (if different from widespread diploid) and tetraploid-specific haplotypes (*y* axis) in each of 14 diploid and 11 tetraploid populations (*x* axis, grouped to lineages and ploidies). Frequencies of minor frequency haplotypes found in either or both ploidies are summed in a gray column. (*B*) A hypothetical maximal variation among haplotypes of meiosis proteins in diploids and tetraploids, quantified by Hamming distances. The diameter of the red and blue circles denotes the full range of potential variability of haplotypes reconstructed by all combinations of AASs among all diploid and tetraploid individuals, respectively. The relative distance of the red and blue circles denotes the genetic distance between the diploid and tetraploid haplotypes. Overlap of both circles suggests that it is plausible that the tetraploid haplotype could have existed within the observed variation in diploids, even if the exact tetraploid haplotype was not found in our diploid sampling. Filled area of the tetraploid circle, nonoverlapping with diploid, represents the tetraploid haplotype space that cannot be explained by, and would not be expected to exist, within extant diploid AAS variation. The upper six proteins show evidence that their tetraploid haplotypes most likely accumulated additional mutations after diploid/tetraploid divergence.

To identify potential targets of positive selection among the set of 78 meiosis proteins, we first scanned sequences for amino acid substitutions (AASs) between 1) all five previously defined diploid lineages (Kolář et al. 2016; Monnahan et al. 2019: *Pannonian*, *Dinaric*, *Baltic*, *Southeastern Carpathian*, and *Western Carpathian*; fig. 1*A* and *B*) and 2) comparing all diploid individuals as a group with the tetraploid lineage, using a subsampled data set of 120 individuals to ensure comparable sample sizes across ploidies and lineages (see Materials and Methods for details, supplementary table S1, Supplementary Material online). We identified outlier differentiated AASs as those exceeding the 99% *F*_ST_ genomewide quantile. We then narrowed this set to those changes predicted to also have functional effects, by selecting the overlap with 1% genomewide outliers identified using the FineMAV method (Szpak et al. 2018; modified to use Grantham and SIFT scores that predict potential functional impact of each AAS, Grantham 1974; Kumar et al. 2009, see Materials and Methods for details). The overlap of *F*_ST_ and FineMAV outliers identified 56 AAS outliers, in seven meiosis proteins, among the pairwise diploid contrasts, and 171 AAS outliers, in 11 meiosis proteins, in the diploid/tetraploid contrast (below, these are termed “candidate selected AASs” and the proteins they occur in as “candidate selected proteins”; supplementary tables S5–S7, Supplementary Material online). We inferred which are the derived variants of each AAS by comparing with three Arabidopsis outgroup species.

To further test for evidence of positive selection on meiosis genes in the tetraploids, we used McDonald–Kreitman test (McDonald and Kreitman 1991, Smith and Eyre-Walker 2002; see Materials and Methods for details). In this method, we calculated alpha, the proportion of divergences driven by positive selection (supplementary table S8, Supplementary Material online). Overall, we found evidence of a significantly increased genomewide proportion of divergence values that show evidence of having been driven by positive selection, between diploids and tetraploids of *A. arenosa* (*α*  =  0.44, *P* value <0.001). Among the candidate meiosis proteins, values of alpha exceeded the neutrality value of zero in all but three cases (supplementary table S8, Supplementary Material online), the exceptions being ZYP1b, ASY3, and SMG7. For five meiosis proteins (PRD3, ASY1, PDS5b, REC8/SYN1, and DYAD), alpha estimates exceeded the genomewide value of 0.44 (*α* between 0.5 and 1, mean = 0.71, *P* values >0.05 due to the low number of divergences), suggesting that these proteins evolved under positive selection. In summary, despite the biases that could arise due to the low divergence between the lineages studied here (Monnahan et al. 2019), the results of McDonald–Kreitman test nevertheless support our FineMAV and *F*_ST_-scan results, supporting the idea that positive selection targeted meiosis proteins during the divergence of diploids and tetraploids.

When analyzing genomewide patterns, Pannonian diploids and tetraploids both had significant excess proportions of meiosis proteins among all candidate positively selected proteins genomewide (*P* = 0.02 and <0.001, respectively, Fisher’s exact test, fig. 1*B* and *C*, supplementary table S7, Supplementary Material online). This was not the case in any other populations or lineages (supplementary table S7, Supplementary Material online). These results show that signatures of positive selection are only prevalent in the two lineages in which we previously identified them and are not a ubiquitous feature of meiotic protein evolution in *A. arenosa*. In addition to confirming previously identified genes, we identified several new candidate meiotic genes that show evidence of having been under positive selection. We discuss these and their functional implications further in supplemental text 1, Supplementary Material online.

We next wished to test if the candidate-selected AASs are likely to affect conserved or potentially functional sites, and whether this propensity differs among lineages. To do this, we first estimated the potential constraint on particular amino acids by calculating pairwise amino acid identity at all candidate-selected AAS sites across the proteomes of 17 Malvidae species with sequenced genomes available (see Materials and Methods). In tetraploids, AASs differentiated from diploids were significantly more likely to affect amino acids that are conserved across plant evolution than AASs that show differentiation among the different diploid lineages (*P* value = 0.002, Wilcoxon rank sum test, fig. 1*D* and supplementary text 2, supplementary fig. S1, Supplementary Material online). Even though multiple meiosis genes also show evidence of positive selection in the Pannonian diploid lineage, in contrast to the tetraploids, this lineage does not differ significantly from other diploid lineages in the proportion of differentiated AASs in meiosis genes that affect conserved sites (*P* value >0.05, *n* = 56, Wilcoxon rank sum test, fig. 1*C*). We also found that the differentiated AASs in tetraploids are predicted to cause secondary protein structure variation (supplementary fig. S2 and supplementary text 3, Supplementary Material online). This is interesting in light of the evidence that 3D structures of meiosis proteins are strongly conserved across even wide evolutionary distances, though the underlying primary sequences can vary substantially even among closely related species (Grishaeva and Bogdanov 2014; Rosenberg and Corbett 2015). These results suggest that tetraploids have both a higher total number of candidate-selected AASs and show evidence that positive selection also targeted more conserved amino acids. This observation supports the hypothesis that greater functional readjustment occurred in the meiotic machinery in the tetraploids than in the diploids.

### Positive Selection in the Tetraploids Acted at Least in Part on De Novo Mutations

The high number of potentially functional amino acid changes in multiple interacting proteins in the tetraploids is striking given their relatively recent origin. We thus hypothesized that at least some of the candidate-selected alleles were likely selected from standing variation that existed in diploids. To explore this, we first examined standing variation present in diploids for amino acid changes that characterize tetraploid alleles. We analyzed 10 of the 11 meiosis proteins that show evidence of positive selection in tetraploids (one, ZYP1a with 26 candidate AASs, was removed due to poor mapping of the gene to the reference genome). We analyzed the full available data set of 105 individuals from 14 genome-resequenced diploid populations (including 23 individuals from the Western Carpathian lineage, the most closely related diploids to the tetraploids, fig. 1*A* and *B*; Arnold et al. 2015; Monnahan et al. 2019). A rarefaction analysis of *A. arenosa* diploids implied that such sampling is sufficient to converge on the full diploid diversity (supplementary fig. S3, Supplementary Material online).

We found that 63% of tetraploid differentiated AASs were not present in any of the diploid individuals sampled (71 out of 113 AASs; however, we note that this is likely an overestimate of the proportion of amino acids absent from the standing variation as some of the variants might be too rare to be sampled, or may have been originally present in diploids, but went extinct after the divergence of the tetraploids). The remaining 42 ploidy-differentiated AASs were found in our diploid samples, indicating a contribution from standing variation. Most of the “standing” AASs occurred in three proteins (PRD3, ZYP1b, and SHOC1; supplementary fig. S4, Supplementary Material online). An additional 32 candidate-selected AASs, not included in the 113 AASs above, showed parallel differentiation in tetraploids and Pannonian diploids (in proteins SMG7, ASY3, and REC8/SYN1, fig. 1*B* and *C*). Whether this pattern is due to incomplete lineage sorting or gene flow between these lineages is not clear.

Since all proteins with evidence of positive selection contain multiple highly differentiated amino acid polymorphisms, we asked if there are instances where full haplotypes of linked candidate-selected AASs exist as standing variation in diploids. We reconstructed the most likely haplotypes across tetraploid-differentiated AASs (supplementary table S6, Supplementary Material online) using allele frequency information complemented with haplotype phasing data in the respective diploid or tetraploid genome assembly (see Materials and Methods). Apart from the SMG7 haplotype, which was found in one population in the Pannonian lineage and in three populations in the Western Carpathian lineage, none of the complete haplotypes predominant in tetraploids were found in any diploid population (fig. 2*A*). Taken together, these findings suggest that although some AASs in each case likely originated as standing variation in diploid populations, additional de novo changes likely accumulated in each of the meiosis genes to generate the extant tetraploid alleles.

The findings above cannot rule out that the full haplotypes were originally present in diploids, but lost after divergence of the tetraploids, or that they were present, but too rare to have been sampled. Thus, we quantified whether an unsampled haplotype allele as different from other diploid variants as the current tetraploid allele is, could plausibly have existed within the range of variation in our sampled portion of the diploid gene pool. If not, this would suggest that additional amino acids likely accumulated postdivergence. To do this, we compared the Hamming distance (which quantifies the number of sites in which diploid and tetraploid alleles differ in nucleotide sequence) to the Hamming diameter of each gene pool (which is the maximum pairwise distance among alleles within a set, see Materials and Methods, Robinson 2003). If the Hamming distance between diploids and tetraploids is lower than the Hamming diameter within diploids, it is considered plausible that the tetraploid haplotype could have existed within the diploid pool of genetic variation, even if not sampled. This was the case for four proteins (fig. 2*B* and supplementary table S9, Supplementary Material online). For six meiotic proteins, however, the tetraploid haplotype was differentiated beyond the diploid variation and thus likely not available within the original pool of standing diploid variation (fig. 2*B* and supplementary table S9, Supplementary Material online). This includes two meiotic axis proteins (ASY1 and ASY3) whose diploid and tetraploid variants have recently been shown to have distinct functional effects in meiosis (Morgan et al 2020).

The proportion of meiosis proteins with likely de novo changes as identified by Hamming distances was only slightly higher than that of the other candidate-selected proteins genomewide (proportion of de novo candidates = 0.60 and 0.52 for meiosis proteins and other proteins genomewide, respectively), suggesting that selection on de novo mutations might be a general feature of positive selection in polyploids. However, meiosis proteins do show an excess relative to other proteins of “de novo” candidate-selected AASs per protein (11.1 for meiosis proteins and 5.4 for other proteins genomewide; *P* = 0.001, Wilcoxon rank sum test), suggesting that meiosis as a process underwent more extensive de novo restructuring than most other processes that show evidence of having been under positive selection in the tetraploid genome.

The above analysis cannot completely rule out allele extinction. However, we note that selection from standing variation followed by allele extinction at multiple independent loci in diploids is not the most parsimonious explanation. We would have to imagine that, six times independently, a standing variant that is more different than any other allele sampled from the present gene pool came under positive selection in the tetraploids and was subsequently lost in diploids. Thus, we believe that although some amino acids characteristic of tetraploid alleles do come from standing variation a considerable fraction of the observed differences accumulated de novo in the tetraploid lineage after divergence.

### The Accumulation of Amino Acid Changes in the Tetraploids

Given that positive selection predominantly from standing variation is an unlikely explanation for the pattern of amino acid divergence in tetraploids, we explored whether rapid protein evolution might be driven by compensatory evolution and coevolution, as previously proposed for autotetraploid *A. arenosa* (Hollister et al. 2012). Compensatory coevolution of interacting proteins can speed the accumulation of novel changes because if a change in one protein causes even a subtle shift in structure or stability, this will lead to selection for compensatory mutations that return the structure or stability of the protein, or an entire complex, to its optimal state (DePristo et al. 2005; Szamecz et al. 2014; Rojas Echenique et al. 2019). Because compensatory mutations have a large mutational target, as any number of amino acid changes can readjust the stability or shape of a protein, they can accumulate rapidly relative to changes that must target particular functional sites (DePristo et al. 2005; Szamecz et al. 2014). Empirical data support this idea, for example, work in bacteria has shown that this kind of compensatory evolution can lead to the rapid accumulation of AASs in groups of interacting proteins (Moura de Sousa et al. 2017). Since meiotic proteins are well known to interact (e.g., Zickler and Kleckner 1999), compensatory evolution and coevolution might be one cause of rapid evolution of amino acid changes (Maisnier-Patin et al. 2002; Davis et al. 2009). Thus, we asked if a process of protein coevolution might have promoted the extensive accumulation of de novo amino acid changes in tetraploids.

**Fig. 3.**
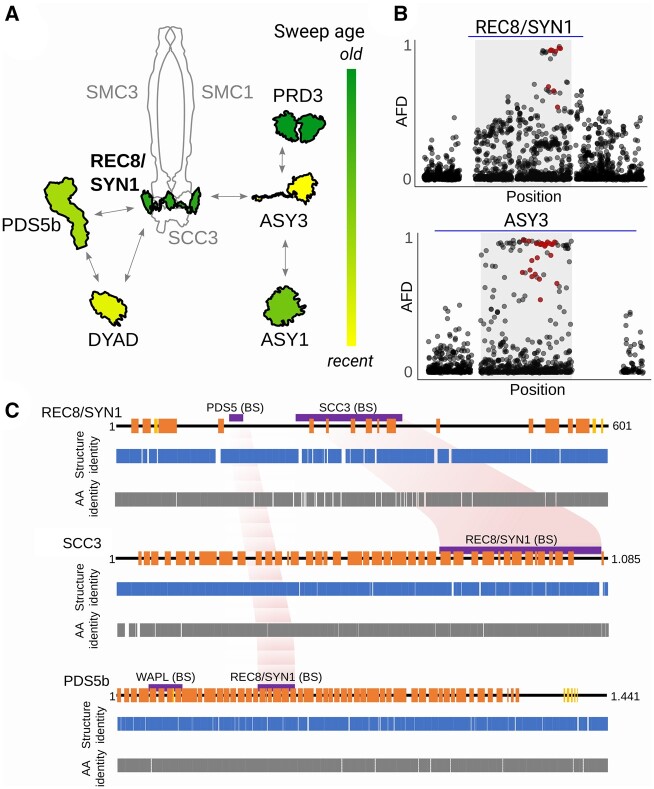
Evidence for meiosis protein coevolution in tetraploids. (*A*) Cartoon of the cohesin complex with associated proteins and variability in relative order of their selection sweeps inferred from haplotype length and number of accumulated SNPs (see Materials and Methods for details). Shown are schemes of candidate protein structures (outlined in black) and other core complex protein structures for illustration (gray). We propose that REC8/SYN1 (bolt) might be the core driver of coevolution as it is the central protein with one of the oldest sweeps. (*B*) Illustrative examples of pattern of allele frequency decay at locus with old (REC8/SYN1) and young (ASY3) selection sweep (as inferred in *A*). Plotted is AFD between diploid and tetraploid individuals for all genic variants in and around the gene. Red dots are candidate AASs identified here; blue line corresponds to 10 kb. (*C*) Coordinated structural changes in protein-binding sites. Cartoons of secondary protein structures from diploid *A. arenosa* meiosis proteins (upper lane; in orange = helix elements, in yellow = sheet elements, and black line = disordered protein regions). The pairwise comparison of predicted secondary protein structures from sequences of diploid and tetraploid *A. arenosa* lineages (middle lane, Structure identity plots) and the identity of their amino acid sequences (lower lane, AA identity plots). Gaps are sites with zero identity. Protein-binding sites and functional domains identified in other eukaryotes are shown as violet bars above the secondary structure plot. Reciprocal structure identity changes in corresponding binding sites of REC8/SYN1 and SCC3 and to lesser degree REC8/SYN1- and PDS5b-binding sites might indicate coevolution of these proteins—highlighted in light red.

We found hints in our data that support the idea that compensatory evolution may contribute to the observed differentiation. First, all six proteins that likely accumulated multiple de novo amino acid changes after divergence of the tetraploids and diploids, are interacting cohesin and axis components, suggesting that changes in one could plausibly affect essential interactions with the others (fig. 3*A*). Second, we examined the relative ages of the selective sweeps (i.e., the likely order in which the tetraploid alleles of the six proteins rose in frequency). Under a coevolution scenario, we might expect positive selection to have acted sequentially on the different cointeracting proteins, rather than all alleles having been targeted at the same time, or that selection acted episodically on each protein as changes occurred in its partners. We estimated the relative sweep age as a ratio of number of SNPs accumulated in the selected haplotype, and its length. For each meiosis protein we counted the number of polymorphisms normalized to the length of the haplotype between first and last candidate positively selected AAS as a proxy for sweep age. The oldest sweeps were inferred to have occurred in PRD3 and REC8/SYN1, followed by ASY1 and PDS5b, with ASY3 and DYAD being the youngest (fig. 3*A* and *B* and supplementary table S10, Supplementary Material online). Age estimates of this sort are error prone (Messer and Neher 2012; Ormond et al. 2016; Smith et al. 2018), but the potentially staggered origin of selected alleles hints that changes in one may have provided a context that favored changes in another (e.g., positive epistasis; Pedruzzi et al. 2018).

We also searched for hints of mechanistic evidence of coevolution, for example, predicted structural differences in binding sites of the candidate proteins. We did this using our diploid and tetraploid genome assemblies, for the subset of proteins with known structures: the cohesin subunit REC8/SYN1, the cohesin regulator PDS5b, and the meiotic axis components ASY1 and ASY3, together with the cohesin component SCC3 (which does not show strong evidence of selection, but where we identified a medium-frequency premature stop codon in tetraploids, supplementary text 2, Supplementary Material online). Using PSIPRED secondary structure predictions, which calculate which of the three local amino acid interactions, helix, sheet or coil elements, are most likely for each position in the amino acid chain, we found clusters of predicted structural changes in the interaction surfaces of REC8/SYN1 and SCC3 and to lesser degree of PDS5b and REC8/SYN1. This finding suggests that these proteins may be coevolving (fig. 3*C*). Whether the structural changes generate novel interaction dynamics, or preserve ancestral ones in the face of other functional changes to the cohesin complex, remains to be tested. Though they are not definitive, the above tests for the expected coevolution of the candidate meiosis proteins are consistent with the idea that coevolution of interacting proteins might indeed have been involved in promoting the accumulation of at least some of the amino acid changes observed.

A potential nonselective explanation for the large number of differentiated AASs in some proteins could be that they arose in single multi-nucleotide mutation (MNM) events, which can give rise to multiple closely linked substitutions in a single instance. A hallmark of MNMs is that substitutions are closely spaced, and commonly also have a significant excess of transversions relative to transitions (Schrider et al. 2011; Harris and Nielsen 2014; Besenbacher et al. 2016). We therefore scanned for these features in genes encoding the candidate-selected meiosis proteins. We found patterns suggestive of MNM events in derived alleles of ASY3 and SMG7, which both had a higher than random proximity of AASs (the median distance = 26 and 46 bp for ASY3 and SMG7, respectively, whereas for other proteins genomewide the distance is 61 bp; *P* < 0.01 in both cases, Wilcoxon rank sum test). Derived alleles in both genes also have a significant excess of transversions relative to transitions compared with genomewide rates (*P* < 0.01, two-sample *z* test). We observed a similar transversion/transition bias in derived alleles of four other proteins in the tetraploid (REC8/SYN1, ASY1, PRD3, and ZYP1a) and REC8/SYN1 in the *Pannonian* diploid (*P* < 0.01, two-sample *z* test), but these latter examples lacked the close spacing of mutations characteristic of MNMs.

## Conclusions

Here we investigated the evolution of meiosis proteins in *A. arenosa* using a rangewide sampling of diploid and tetraploid lineages (Monnahan et al. 2019). Since many meiosis proteins are thought to evolve rapidly (Heyting 1996; Kumar et al. 2010; Grishaeva and Bogdanov 2014; Baker et al. 2017; Brand et al. 2019), we reasoned that signals of positive selection might be common, and therefore not unique to the two *A. arenosa* lineages where they were found previously (Hollister et al. 2012; Yant et al. 2013; Wright et al. 2015). However, we found from sampling four additional lineages that strongly differentiated AASs were found almost exclusively in these two lineages (the tetraploid and the *Pannonian* diploid), suggesting that positive selection on these genes is situational and not ubiquitous. The pattern in the tetraploid lineage is especially striking; despite its tender evolutionary age (∼20,000–30,000 generations; Arnold et al. 2015; Monnahan et al. 2019), it has the largest number of proteins with excessive differentiation, the largest number of differentiated AASs, a higher proportion of AASs in conserved sites, amino acid changes occurring in the interaction surfaces of the meiotic cohesin alpha-kleisin (REC8/SYN1) and its interacting partners, and predicted structural shifts. Thus, the shift in the tetraploid meiotic machinery appears to be far more substantial than what occurred in any diploid lineage of *A. arenosa* including the Pannonian lineage. This fits with the idea that genome duplication is an especially strong challenge for meiosis, likely necessitating a rapid evolutionary response (Bomblies et al. 2015, 2016).

We also find evidence that a considerable proportion of amino acids that are differentiated between the diploid and tetraploid lineages likely arose de novo on alleles that already contained some polymorphisms that preexisted as standing variation. This likely high contribution from de novo variation might come as a surprise, given the theoretical prediction and empirical evidence that rapid evolution is greatly facilitated by the availability of preexisting genetic variation (Jones et al. 2012; Olson-Manning et al. 2012; Ralph and Coop 2015; Van Belleghem et al. 2018; Alves et al. 2019; Haenel et al. 2019; Lai et al. 2019; Oziolor et al. 2019; Thompson et al. 2019). We suggest three nonmutually exclusive explanations for why this might be: 1) Meiosis is a conserved multiprotein process whose need for restructuring after polyploidization (Comai 2005; Bomblies and Madlung 2014) requires variants that are perhaps deleterious in the diploid background. 2) The considerable contribution of novel mutation to rapid adaptation may be a more common feature of autopolyploid evolution, perhaps due to their higher effective population size and/or lower homozygosity (Parisod et al. 2010) or the sudden novel physiological context of polyploids (Doyle and Coate 2019; Bomblies 2020). 3) Empirical literature may be biased toward reports of adaptation from standing variation (Barrett and Schluter 2008), as it is easier to detect a presence of genetic variants than to exclude it.

In summary, our study supports the idea that both standing and de novo variation may be important sources of adaptive variants in multiple interacting meiosis proteins in autotetraploid *A. arenosa*. It will be interesting to see whether this is a particularly prominent feature of polyploid evolution, or a more common pattern for the evolutionary modification of conserved multiprotein processes that occurs when populations must adapt to sudden novel circumstances that challenge these processes.

## Materials and Methods

### Data Sets

#### Sampling and Population Genetic Structure of the Genomic Data Set

To study the evolution of meiosis proteins in both diploid and tetraploid *Arabidopsis arenosa* populations, we reanalyzed a rangewide genomic data set previously described in (Monnahan et al. 2019). This data set originally consists of sequences from 15 diploid and 25 tetraploid genome resequenced *A. arenosa* populations (287 individuals, seven individuals per population on average, supplementary table S1, Supplementary Material online). We first aligned the short-read sequences to the *Arabidopsis lyrata* version 2 (LyV2) reference genome (Hu et al. 2011), called variants and filtered as previously (Monnahan et al. 2019) using the Genome Analysis Toolkit (GATK 3.5 and 3.6, McKenna et al. 2010) and finally called SNPs with GATK HaplotypeCaller. For most analyses described below (except where noted), we used a subset of the full data set consisting of 80 diploid individuals (16 samples with the highest depth of coverage of sequences from each of the five major lineages) and 40 tetraploid individuals from populations unaffected by secondary introgression from diploid lineages (i.e., sampling from C European, Alpine, and Swabian lineages as defined in Monnahan et al. (2019). Such subsampling gave us a balanced number of 160 high-quality haploid genomes of each ploidy suitable for unbiased scans for positive selection, which was also unaffected by later unidirectional interploidy introgression (supplementary table S1, Supplementary Material online). Finally, we filtered each subsampled data set for genotype read depth >8 and maximum fraction of missing genotypes <0.5 in each lineage to be confident about the variant calling.

We used our total diploid sampling (105 individuals, supplementary able S1, Supplementary Material online) in a separate analysis aimed to screen for standing variation of tetraploid alleles in the total diploid sample (fig. 2 and supplementary fig. S4, Supplementary Material online). This yielded the total number of 145 resequenced individuals used throughout our analyses.

To avoid polarization toward a single reference species genome, we repolarized the variants using a collection of individuals across three closely related diploid Arabidopsis species, European *A. lyrata*, *A. croatica*, and *A. halleri*, following procedure described in Monnahan et al. (2019). We further confirmed the repolarization using frequencies of the variants across the data set (considering the minor frequency allele overall as derived).

We calculated genomewide nucleotide diversity (*π*) and Tajima’s *D* (Tajima 1989) for each lineage, all diploids and all tetraploids using putatively neutral 4-fold degenerate sites. In agreement with the previous study (Monnahan et al. 2019), the per-population genomewide synonymous diversity (*π*) was similar between ploidies (*π* values ranging between 0.028 and 0.032 in five diploid lineages, 0.036 for all diploids and 0.034 for tetraploids, supplementary table S4, Supplementary Material online) and total range of Tajima’s *D* over synonymous sites (−0.34 to +0.34, supplementary table S4, Supplementary Material online) was far from the accepted threshold of nonneutrality (±2; Tajima 1989). Calculations were performed using python3 ScanTools pipeline (github.com/mbohutinska/ScanTools_ProtEvol), a modification of ScanTools, a toolset specifically designed to analyze diploid–autotetraploid data sets.

#### Novel Diploid and Tetraploid Genome Assemblies

We created two *A. arenosa* draft reference assemblies, to investigate the haplotypes of meiosis proteins and differences in secondary structure prediction in a sufficient detail, as well as to remap the areas in the *A. lyrata* genome, where the *A. arenosa* reads did not map well (7 out of the 78 loci, see the next section for details). We assembled genome of one diploid (from *Western Carpathian* population SNO) and one tetraploid individual (population TBG). The diploid assembly is also described in (Liu et al. 2020), but we include it here for completeness.

First, fresh leaf material was sent to Earlham Institute, where DNA was extracted using the BioNano plant protocol from the tetraploid *A. arenosa* and using CTAB DNA extraction protocol from *A. arenosa* diploid (as in Paajanen et al. 2019). Second, to construct the 10× library, DNA material was diluted to 0.5 ng/μl with EB (Qiagen) and checked with a QuBit Flourometer 2.0 (Invitrogen) using the QuBit dsDNA HS Assay kit. The Chromium User Guide was followed as per the manufacturer’s instructions (10× Genomics, CG00043, Rev A). The final library was quantified using quantitative polymerase chain reaction (qPCR, KAPA Library Quant kit [Illumina], ABI Prism qPCR Mix, Kapa Biosystems). Sizing of the library fragments were checked using a Bioanalyzer (High Sensitivity DNA Reagents, Agilent). Samples were pooled based on the molarities calculated using the two QC measurements. The library was clustered at 8 pM with a 1% spike in of PhiX library (Illumina). The pool was run on a HiSeq2500 150 bp Rapid Run V2 mode (Illumina). The following run metrics were applied: Read 1: 250 cycles, Index 1: 8 cycles, Index 2: 0 cycles, and Read 2: 250 cycles.

Sample TBG was sequenced on HiSeq2500 Rapid Run V2 mode (Illumina, on 150-bp sequences). About 58.49 M (121.71 M) reads were created. These were assembled on Supernova 2.0.0 giving raw coverage 27.66× and effective coverage 22.07×. The molecule length was 57.19 kb. The assembly size, counting only scaffolds longer than 10 kb was 58.84 Mb, and the Scaffold N50 was 33.92 kb.

Sample SNO was sequenced on HiSeq2500 Rapid Run V2 mode (Illumina, on 150-bp sequences). About 82.10 M reads were created. These were assembled on Supernova 2.0.0 giving raw coverage 57.91× and effective coverage 45.30×. The molecule length was 26.58 Kb. The assembly size, counting only scaffolds longer than 10 kb was 127.02 Mb and the Scaffold N50 was 2.19 Mb (supplementary table S2, Supplementary Material online).

We analyzed the gene content using BUSCO, and the results showed that the gene space of the diploid *A. arenosa* assembly was nearly complete with 97.5% of the plant specific BUSCOs present and 1.4% missing completely. Of these, 4.7% were duplicate copies.

With the tetraploid *A. arenosa* assembly, we captured 98.5% of the core plant genes and had 1.3% missing. Since the plant is a tetraploid, the rate of duplicate genes was high in the assembly, and total of 82.8% of the core plant genes were found as duplicates. This is not surprising, especially since the plant was from the TBG population that is in the railway lineage and hence shows secondary admixture from a diploid *A. arenosa* lineage (Monnahan et al. 2019). Thus when working with the TBG fragmented assembly, we always checked the variation among all diploid and nonadmixed tetraploid populations for confirmation which of the two cooccurring haplotypes is dominating our tetraploid sampling.

### Detecting Signatures of Positive Selection Acting on Meiosis Proteins

#### Meiosis Protein Identification, Processing, and Annotation

We annotated each SNP in the genomewide data set and assigned it to a gene using SnpEff 4.3 (Cingolani et al. 2012) and following *A. lyrata* version 2 genome annotation (Rawat et al. 2015). Annotated variants genomewide were extracted from vcf format to table using SnpSift, part of SnpEff 4.3, with flags “CHROM POS REF ALT AC AN ‘ANN[*].HGVS_P’” and these tables were used as the basis for the subsequent analysis of positive selection. Next, we identified a list of 78 proteins related to meiosis was based on Yant et al. (2013) and updated by searching PID, AraNet (Probabilistic Functional Gene Network of *A. thaliana*) and *A. thaliana* orthologs in TAIR database (Berardini et al. 2015) and using the list of meiosis proteins from (Sánchez-Morán et al. 2005). ZYP1A, which is not present in the *A. lyrata* version 2 annotation, was added manually based on gene model available from the previous study (Yant et al. 2013). We assigned it with ID AL1G35725 to place it in the correct order into the reference .gff3 file. We further validated that the meiosis genes were expressed in *A. arenosa* using an available RNASeq data set (supplementary text 4, Supplementary Material online).

We found seven meiosis genes (SHOC1, SCC1, SCC2, SCC3, SCC4, MSH4, and SMC6A), where duplicated regions mapped to the same reference loci or where the reads were mismapped when aligning to the *A. lyrata* reference (Hu et al. 2011). To overcome this problem, we realigned these loci separately to our own *A. arenosa* diploid reference. To do so, we took the *A. arenosa* reference sequence and found the *A. lyrata g*enes in the assembly using bwa 0.7.12 (Li 2013). We extracted 20 kb upstream and downstream from the gene and created a new reference with just these seven genes. Then we mapped the raw reads from each of the 291 samples back to this reference, following the same procedure which we used for mapping to *A. lyrata*. The heterozygosity and coverage of newly remapped genes stayed within the genomewide average. The commands that were used are available at (github.com/paajanen/meiosis_protein_evolution/). We built a separate *A. arenosa* database for these mismapped genes using our *A. arenosa* reference sequence and gff3 files made manually based on *A. lyrata* V2 gff3 using Geneious 11.0.3. The SnpEff analyses then followed the above outlined procedure and the total list of all 78 meiosis genes was analyzed jointly hereafter.

#### Scans for Positive Selection with Likely Functional Consequences Acting on Meiosis Proteins

To infer candidate AASs within our data set of 78 meiosis genes, highly differentiated between lineages and with likely impact on protein function, we combined a differentiation-based positive selection scan (*F*_ST_, Hudson et al. 1992) with genome scanning method accounting for theoretical functional consequence of each AAS (modified FineMAV, Szpak et al. 2018). Both methods are well suited to infer signatures of recent (within species) positive selection (Oleksyk et al. 2010; Vitti et al. 2013). We used both approaches based on population allele frequencies, allowing joint analysis of diploid and autopolyploid populations. We screened for positive selection 1) among the five diploid lineages (fig. 1*A*) and 2) between all diploids and tetraploids. We considered only AASs that were outliers in both selection scans as putative positive selection candidates. For these analyses, we worked with six lineages in total, covering a full known distribution range of *A. arenosa* (fig. 1*A* and *B*; Kolář et al. 2016; Monnahan et al. 2019): *Pannonian*, *Dinaric*, *Baltic*, *Southeastern Carpathian*, and *Western Carpathian* (diploid lineages, subsampled to 32 chromosomes each) and tetraploid (subsampled to 160 chromosomes and contrasted to the sum of all 160 diploid chromosomes). A reanalysis of diploid–tetraploid selection scans using 16 diploid and 16 tetraploid individuals (comparable with the sample size of diploid) did not yield qualitatively different results. First, for each lineage pair, we calculated *F*_ST_ for all nonsynonymous SNPs (i.e., AASs) across the 78 meiosis proteins. We used Hudson’s *F*_ST_ estimator, which is suitable for a single variant calculations (Bhatia et al. 2013). Next, we calculated distribution of *F*_ST_ over all synonymous (i.e., putatively functionally neutral) SNPs genomewide. We used the 99th quantile of this “neutral” distribution as a threshold for identification of outlier AASs. The neutral synonymous *F*_ST_ quantiles did not differ significantly from those derived from nonsynonymous SNPs (supplementary table S11, Supplementary Material online, Wilcoxon rank sum test, *W* = 69.5, *P* value = 0.58, *n* = 11). However, the quantile values were consistently slightly lower for nonsynonymous SNPs (supplementary table S11, Supplementary Material online), making the use of synonymous *F*_ST_ quantiles more conservative. All calculations were performed using ScanTools_ProtEvol, and custom R scripts (github.com/mbohutinska/ProtEvol/).

Second, we adopted the Fine-Mapping of Adaptive Variation (FineMAV, Szpak et al. 2018) and modified it to fit the resources available for *A. lyrata* reference genome. Specifically, we replaced CADD, the functional score available for human reference (Szpak et al. 2018; Rentzsch et al. 2019), by 1) the Grantham score (Grantham 1974), which is a purely theoretical AAS value, encoded in the Grantham matrix, where each element shows the differences of physicochemical properties between two amino acids and 2) the SIFT annotation score (Kumar et al. 2009), which estimated the effect of amino acid change based on sequence homology across available reference sequences and physical properties of amino acids. To estimate the SIFT scores specifically for our data set, we created a SIFT annotation of our vcf-file using *A. lyrata* database v.1.0.23 from SIFT website (https://sift.bii.a-star.edu.sg/sift4g/, last accessed February 3, 2021). The annotation was done using SIFT4G algorithm (command java -jar SIFT4G_Annotator_v2.4.jar -c -i input.vcf -d ./Lyrata_db/v.1.0.23/-r annotated). We rescaled the SIFT score to be 1 when it is most deleterious and 0 when it is most tolerated. Next, we estimated the population genetic component of FineMAV (see Szpak et al. 2018 for details on calculations) using allele frequency information at each site (considering minor frequency allele as derived) and DAP parameter of 3.5. Finally, for each AAS, we assigned Grantham scores and SIFT scores, together with population genetic component of FineMAV, using a custom scripts in Python 2.7.10 and the Biopython 1.69 package. By rescaling the SIFT scores, we ensured that for both functional score, higher value indicate more likely impact of the AASs to the protein function. Finally, we identified the overlap of top 1% outlier AASs identified in the FineMAV analysis with SIFT scores and with Grantham scores and considered these double outlier AASs as a final candidate identified in FineMAV analysis. All the calculations were performed using code available at (github.com/paajanen/meiosis_protein_evolution).

We note that the SIFT database was developed for *A. lyrata* annotation version 1, and do not contain all meiosis proteins from our list. Thus, we did not obtain any SIFT score for SCC3, MSH4, SMC6A, and ZYP1a and we only considered Grantham scores for them (supplementary tables S5 and S6, Supplementary Material online).

Finally, we controlled for the presence of differentiated indel variants in all candidate meiosis proteins by inspecting their alignment files of the RNA-Seq mapping and screening their gene sequences in the newly generated diploid and tetraploid draft assemblies. We identified only three indel variants differentiated between diploids and tetraploids and neither of them was a frameshift mutation affecting any of our candidate AASs. Thus, the indel variants should not affect the interpretations of our SNP-based selection scans.

Finally, to further assess selection acting on meiotic proteins, we conducted a McDonald–Kreitman test, which is a powerful approach for detecting selection in proteins (McDonald and Kreitman 1991, Smith and Eyre-Walker 2002). We calculated alpha, which quantifies the proportion of divergence driven by positive selection and is defined as *α* = 1 − (D_S_P_N_)/(D_N_P_S_), where D_S_ and D_N_ are the numbers synonymous and nonsynonymous substitutions per gene, respectively, and P_S_ and P_N_ are the numbers of synonymous and nonsynonymous polymorphisms per gene. The divergence between diploids and tetraploids of *A. arenosa* is too recent to satisfy the assumption of fixation of nucleotide substitutions within species. We thus estimated nucleotide divergence values (D_S_, D_N_) using the upper 1% outliers of allele frequency differences (AFD) between diploids and tetraploids (upper 1% AFD outlier treshold = 0.53). It has also been suggested that it is important to exclude rare polymorphisms to minimize the impact of slightly deleterious mutations on the estimate of adaptive evolution (Charlesworth and Eyre-Walker 2008). Thus, we excluded variants with overall allele frequency lower than 0.15 (following Fay et al. 2001; Zhang 2005).

#### Ortholog Search and Analysis of Evolutionary Conservation of Candidate AASs

To examine the tendency of candidate AASs to affect conserved sites, we compared levels of pairwise alignment identity (PAI, mean pairwise identity over all pairs in the alignment column) of the 78 meiosis protein sequences across the proteomes of 17 Malvidae reference genomes. To do so, we downloaded *A. lyrata* sequences of the meiosis proteins from Phytozome12.1 database (www.phytozome.jgi.doe.gov, last accessed August 7, 2018) and used as query sequences to identify orthologs of 17 Malvidae species proteomes. Species included in the search were *Arabidopsis halleri*, *A. thaliana*, *Boechera stricta*, *Capsella grandiflora*, *Capsella rubella*, *Eutrema salsugineum*, *Brassica rapa*, *Brassica oleracea*, *Populus trichocarpa*, *Salix purpurea*, *Theobroma cacao*, *Manihot esculenta*, *Gossypium raimondii*, *Carica papaya*, *Citrus clementina*, *Citrus sinensis*, and *Linum usitatissimum*. We performed searches using the BlastP program in Phytozome with proteome as target type, e-threshold −1 and BLOSUM62 comparison matrix. In case of identification of multiple orthologs (i.e., multiple hits for the same species), only the ortholog with the lowest *e*-value was considered. The number of sequences in protein alignments ranged 13–17 (16.5 on average, supplementary table S12, Supplementary Material online). We aligned protein sequences of all identified orthologs using MUSCLE as implemented in Geneious v11 (Kearse et al. 2012), with default settings (UPGMB clustering method, terminal gaps full penalty, gap open score −1, window size five). PAI was extracted for each reference (*A. lyrata*) amino acid and we tested the difference in the PAI of diploid and tetraploid candidate AASs sites using Wilcoxon rank sum test (R package stats, R Core Team 2018).

### Distinguishing between Positive Selection on De Novo Mutations and Standing Variation

We used a three-step procedure to distinguish whether positive selection in each candidate meiosis protein likely acted on de novo mutations or standing variation: 1) search for the presence of candidate tetraploid-differentiated AASs across full sampling of individuals from all known diploid lineages of *A. arenosa*, 2) search for the presence of tetraploid-differentiated haplotypes across these diploid individuals, and 3) study of uniqueness of tetraploid haplotypes by comparing their differentiation from diploids to their overall diploid diversity.

In order to conclude that positive selection in a candidate meiosis protein likely acted on de novo variation, we requested that all three of these criteria pointed toward de novo origin in tetraploids; that is, that at least some of its candidate tetraploid-differentiated AASs were not found in any diploid individual, the complete tetraploid haplotype was not find in any diploid individual, and the tetraploid haplotype divergence from the diploid exceeds the overall diploid diversity (diploid–tetraploid Hamming distance exceeding diploid Hamming diameter).

#### The Presence of Candidate Tetraploid-Differentiated AASs in Diploid Lineages

To identify possible standing variation for the tetraploid alleles, we searched for the presence of each candidate tetraploid-differentiated AASs in diploids. We analyzed the full sampling of all 105 individuals from the 14 diploid populations, covering all known lineages of *A. arenosa* (fig. 1*A*, Kolář et al. 2016; Monnahan et al. 2019). The rarefaction analysis implies that our sample of 105 individuals is sufficient to converge on the true diversity of *A. arenosa* diploids. In fact, the rarefaction curve (supplementary fig. S3, Supplementary Material online) suggests that as little as 40 diploid individuals sampled across the *A. arenosa* species range would be enough to cover most of its diploid diversity.

#### Reconstructed Haplotypes across Linked Candidate AASs

To search for the presence of tetraploid haplotypes in diploids, we reconstructed lineage-specific haplotypes and their allele frequencies across the sets of linked candidate AASs within each candidate protein in tetraploids (supplementary table S6, Supplementary Material online). We used this simplified procedure as we were not able to use standard phasing procedures reliably, due to the fact that we were using short reads and working with tetraploids (Kyriakidou et al. 2018).

For each protein, with *n* candidate AAS sites in the data set of 145 individuals consisting of 105 diploids and 40 tetraploids, we defined $M_{i}$ to be the major allele frequency at the candidate AAS site *i*, given that the sample consists of 160 tetraploid haplotypes, and 210 diploid haplotypes, this major allele frequency is going to be dominated by the diploid haplotype, thus we define the ancestral (i.e., diploid) haplotype allele frequency as $HAF_{d}=min\{M_{i}\}$, and consequently, we define the derived (i.e., tetraploid) HAF as $HAF_{a}=1-max\{M_{i}\}$. We further define the frequency of all other haplotypes, which result from recombination of the two previous, as $HAF_{r}=1-HAF_{a}-HAF_{d}$.

We checked for reliability of our approach by extracting haplotypes from our diploid and tetraploid assemblies. Extracted diploid and tetraploid haplotypes of candidate meiosis proteins were consistent with the diploid and tetraploid haplotypes combined based on the allele frequencies at candidate AAS sites.

For all calculations, we used our in-house R script (github.com/mbohutinska/ProtEvol).

#### Hamming Distance and Diameter

In order to study the uniqueness of the tetraploid haplotypes, we defined a measure based on maximum pairwise Hamming distance within a sample (Robinson 2003). In our setting, the Hamming distance compares distances between genotypes, for diploids we first define a distance between alleles such that if the genotypes of two different plants at a given loci is AA aa or aa AA, the genotypic distance is 1, and for pairs AA Aa, Aa aa, Aa Aa, Aa AA, AA AA, aa Aa, the genotypic distance is 0. For tetraploids, we define the genotypic distance to be 1 if the pairs of genotypes are AAAA aaaa, AAAa aaaa, AAAA Aaaa, aaaA AAAA, aaaa AAAa, aaaa AAAA and 0 otherwise. For diploid/tetraploid comparison, we define the genotypic distance to be 1 for the pairs AA aaaa, AA Aaaa, aa AAAA, aa AAAa and 0 otherwise.

The Hamming distance is the sum over all positions that are different. The maximum pairwise numbers are called the Hamming diameter. If the Hamming distance between diploids and tetraploids exceeds Hamming diameter within diploids, it becomes plausible that the AASs forming the tetraploid haplotypes originated de novo. This is a conservative indication of possible de novo origin of the tetraploid haplotype, as the fact that all the AASs forming the tetraploid haplotype are standing in the diploids does not imply that the complete tetraploid haplotype preexisted in any diploid individual.

The code used for the calculations is available in github (https://github.com/paajanen/meiosis_protein_evolution/).

### Compensatory Evolution and Coevolution

#### Timing of Sweeps Using the Haplotype Information

Assuming hard sweep, the sweeping allele initially clears variation on the swept haplotype in a population, but over time, new variants accumulate. In addition, recombination causes the length of swept haplotypes to decline over time (Ormond et al. 2016; Stephan 2019). We thus combined these two metrics to infer the relative age of selection sweeps within the subset of six candidate meiosis proteins with signs of de novo origin of the selected haplotype. For each meiosis protein, we used the haplotype interval between first and last candidate AASs. We took the length of the haplotype in base pairs and measured how many new mutations had appeared in the set of the tetraploid genomes between the first and the last candidate AAS, excluding the candidate AASs, and normalized this count by the length of the haplotype. Finally, we considered the protein with the highest proportion of accumulated mutations in the selected haplotype as the oldest. Note that the short-read population genomic tetraploid data did not allow for reliable phasing so we could not use any method relying on haplotype length decay across individuals.

#### Secondary Structure Prediction in a Subset of Candidate Meiosis Proteins

Coding sequences of candidate meiotic genes were extracted from our diploid and tetraploid *A. arenosa* reference genomes. Open-reading frames were translated into amino acid sequences using Geneious v11 (Kearse et al. 2012). The presence of characteristic amino acid polymorphisms found in this study, conserved in diploid and tetraploid *A. arenosa*, could be confirmed in the extracted sequences. The online tool PSIPRED was used to predict secondary protein structures (Jones 1999; bioinf.cs.ucl.ac.uk/psipred/, last accessed February 3, 2021). The PSIPRED algorithm calculates the likelihood of local amino acid interactions including coil (C; disordered), helix (H), or sheet structures (E) for every amino acid position. The folding of amino acid chains into 3D structures is influenced by local forces (interactions between close amino acid residues, connected neighbors), which determine the secondary structure, and nonlocal forces (topological neighbors), which lead to the tertiary structure. The PSIPRED algorithm includes two feed-forward neural networks that perform an analysis of the output of PSI-Blast (position-specific iterated-Blast), which in turn is based on an alignment of multiple protein sequences. To compare secondary structures of meiotic proteins with each other, sequences of secondary structures from diploid and tetraploid *A. arenosa* were pairwise aligned using the Geneious alignment tool with default settings. Structure identity scores (0; 1) were extracted and plotted together with the identities of the amino acid sequences. Binding sites were identified by literature search: PDS5b-binding site in REC8/SYN1 (Muir et al. 2016), SCC3-binding site in REC8/SYN1- and REC8/SYN1-binding sites in SCC3 (Roig et al. 2014; Orgil et al. 2015), and WAPL-binding site in PDS5b (Ouyang et al. 2016).

### Evidence for MNMs

We observed that in some of our candidate proteins, the candidate AASs were <20 bp apart (supplementary tables S5 and S6, Supplementary Material online), a common rough way how to define MNMs in human or *Drosophila* (Schrider et al. 2011; Besenbacher et al. 2016). Thus, we tested if distances between our candidate AASs in tetraploids were significantly shorter than distances between sites harboring missense SNPs in genes genomewide. We repeated the analysis over tetraploid individuals from the subsampled data set and the results were consistent, so we report results for the individual with the highest coverage SWA_002_1. We used Wilcoxon rank sum test to compare distances between candidate AASs within candidate meiosis proteins and any SNPs genomewide (R package stats, R Core Team 2018).

Another evidence for MNMs is a significant excess of transversions relative to transitions compared with genomewide counts. Thus, for each SNP, we determined if it is a transition or transversion using SnpEff (Cingolani et al. 2012) and tested for excess of transversions relative to transitions in our candidate proteins compared with genomewide counts using *z* test (R package stats, R Core Team 2018).

## Code Availability

Custom scripts used in this paper are available at the following github repositories https://github.com/mbohutinska/ProtEvol, https://github.com/mbohutinska/ScanTools_ProtEvol, https://github.com/paajanen/meiosis_protein_evolution.

## Supplementary Material

Supplementary data are available at *Molecular Biology and Evolution* online.

## Supplementary Material

msab001_Supplementary_Data
