# Supplementary material for: De Novo Mutation and Rapid Protein (Co-)evolution during Meiotic Adaptation in Arabidopsis arenosa
Source: Mol Biol Evol. 2021 Jan 27;38(5):1980–94. doi: 10.1093/molbev/msab001 (PMC8097281; doi:10.1093/molbev/msab001)
Supplement: msab001_Supplementary_Data [file msab001_supplementary_data.zip › Bohutinska_et_al_supplements_fin_revised3.pdf]

SUPPLEMENTARY MATERIALS

De-novo mutation and rapid protein (co-)evolution during meiotic adaptation in Arabidopsis arenosa

Magdalena Bohutínská, Vinzenz Handrick, Levi Yant, Roswitha Schmickl, Filip Kolář, Kirsten Bomblies, Pirita Paajanen

Supplemental Notes

Supplementary Text 1: Newly discovered selection candidates refine our understanding of the response of meiosis to genome doubling

Tetraploids: In the tetraploid lineage, we found four previously unreported proteins that are strongly differentiated between tetraploid and diploid *A. arenosa*, expanding the list of meiosis proteins that might be potential targets of selection in tetraploids. The proteins we previously identified as being under selection in the tetraploids include members of the cohesin complex (SYN1, PDS5b, SMC3, SCC2), as well as structural components of the long linear chromosome axes (ASY1, ASY3) and synaptonemal complex (ZYP1a, ZYP1b) (Hollister et al. 2012; Yant et al. 2013; Wright et al. 2015). Our hypothesis is that these genes are under selection primarily for their roles in chromosome organization, pairing and homologous recombination, features that are critical for the stability of polyploid meiosis (Bomblies et al. 2016). One of the major challenges for polyploids is meiosis, one of the main features of which, in polyploids, is the formation of multivalent chromosome associations among the available homologs (Comai 2005; Stenberg and Saura 2013; Bomblies and Madlung 2014; Bomblies et al. 2016). For two of the proteins under selection in *A. arenosa*, the axis components ASY1 and ASY3, we have confirmed that the derived alleles have functional effect on meiosis, including decreasing axis length and reducing the number of multivalents that form in meiosis (Morgan et al. 2020). An additional protein under selection is PRD3 (Yant et al. 2013; Wright et al. 2015), a homolog of yeast *Mer2*, which interacts with axis proteins to bring the double strand break complex from the loops, where it makes the breaks in the DNA, to the axis, where recombination events are designated and mature (Tessé et al. 2017). All of this paints a picture that protein complexes surrounding cohesin and interacting with it, are targets of evolutionary modification in tetraploid *A. arenosa*, and that at least some have measurable functional consequences in tetraploid meiosis.

Three of the newly identified candidates for selection in tetraploids (SHOC1, SCC4 and DYAD/SWITCH1) help flesh out this picture. SCC4 is part of a complex (made up of SCC2 and SCC4) that loads cohesin onto chromatin, but it also plays critical roles in the regulation of cohesin occupancy on chromatin (Wells et al. 2017). We previously identified the gene encoding SCC2 as a candidate for selection in tetraploids (Hollister et al. 2012). SCC4 could not previously have been identified because in read mapping to the *A. lyrata* reference genome in our previous studies (Hollister et al. 2012; Yant et al. 2013) did not resolve well and was thus missing from our analyses. Using our newly generated 10X Genomics reference genomes we were able to resolve this locus here. DYAD/SWITCH1, like the cohesin complex, is important in chromatin structure and the establishment of sister chromatid cohesion (Yang et al. 2019). Recently, it was shown that SWITCH1/DYAD functions as an antagonist of WAPL, a protein that associates directly with the cohesin ring and acts to remove cohesin from DNA (Yang et al. 2019). Its role in cohesin regulation, and direct association with the cohesin complex itself, places SWITCH1/DYAD firmly within previously identified functions under selection in tetraploid *A. arenosa*, and underscores that modifying the cohesin complex likely plays a crucial role in polyploid evolution, at least in *A. arenosa*. Interestingly, cohesin subunits, including REC8/SYN1, have been implicated in recombination rate variation as well (Sandor et al. 2012; Bauer et al. 2013; Johnston et al. 2016).

SHOC1 is involved in DNA repair and recombination. It is an XPF endonuclease required for the formation of class I crossovers in Arabidopsis (Macaisne et al. 2008). Mutants for SHOC1 show a drastic reduction in crossover number, and the protein is thought to be involved in the maturation of crossover-designated DNA recombination intermediates and functions in the same CO formation pathway as the ZMM proteins MSH5, MSH4 and ZIP4 (Macaisne et al. 2008; Macaisne et al. 2011). Homologs are detected in a wide range of other eukaryotes (Macaisne et al. 2008) and their function is likely conserved, for example in mice a homolog of SHOC1 is also critical for the formation of recombination intermediates (Guiraldelli et al. 2018). Relative to other proteins we have found to be under selection and/or differentiated in *A. arenosa* tetraploids, SHOC1 is functionally distinct in being directly involved in the maturation of crossovers. However, it is an interesting candidate, in that it can affect crossover frequency, a trait known to differ between diploid and tetraploid *A. arenosa* (Comai 2005; Yant et al. 2013) and likely critical for polyploid meiotic stability (Bomblies et al. 2016).

Diploids: We previously found evidence that the meiotic kleisin REC8/SYN1 as well as the axis protein ASY3, with which cohesin interacts, are under selection in a diploid lineage from

the Pannonian basin in Hungary (Wright et al. 2015, fig. 1A). Here we again found evidence of differentiation in these proteins, but also found an additional cohesin interactor, PDS5E, that also showed evidence of differentiation. PDS5 proteins interact with the cohesin ring structure, and function as WAPL antagonists to regulate the persistence of cohesin on chromosomes (Tong and Skibbens 2015). In Arabidopsis, there are five PDS5-like proteins, but only two of them, PDS5a and PDS5b, are full length with respect to those found in other eukaryotes (Pradillo et al. 2015). What the role is of the shorter versions of PDS5, including PDS5e, is unclear, so it is too early to speculate whether the differentiation in PDS5e in Pannonian diploids is related to that in REC8/SYN1 or not.

In the *Scarp* and *Baltic* diploids, there is some evidence for differentiation also in PDS5e, but also in SMC6b, and RMI1, both of which are involved in DNA repair (Verver et al. 2016; Lambing et al. 2017). These proteins are not necessarily strictly meiotic, though they may play roles in meiotic recombination as well (Lambing et al. 2017).

Convergence: Three candidate selection targets appeared in both the *Pannonian* diploids and tetraploids (ASY3, REC8/SYN1 and SMG7) (significant test for convergence; $p < 0.001$, Fisher’s exact test). The derived amino acid substitutions (AASs) in ASY3 and REC8/SYN1 were located at distinct position within the protein sequence in the *Pannonian* diploids and tetraploids, suggesting two independent selective events targeting the same proteins as previously proposed (Wright et al. 2015). The pattern in SMG7 was different, with *Pannonian* and *Western Carpathian* diploid and tetraploid variants clustering together (fig. 1C), suggesting that they might have undergone selection deeper in the history of *A. arenosa*, or that the putatively selected alleles spread among lineages by subsequent gene flow (fig. 1C). SMG7 is functionally potentially quite interesting, but distinct from the previously identified proteins: it plays an important role in progression of the meiotic cell cycle (Riehs et al. 2008; Bulankova et al. 2010). Why it might be under selection is not clear. Perhaps it is important to modulate timing of the meiotic cell cycle in different climates, or in polyploids.

Supplementary Text 2: The level of evolutionary conservation and predicted functional effect of candidate amino acid substitutions.

Functionally critical amino acids in proteins are expected to be more conserved throughout the evolution. Thus, we examined the tendency of candidate amino acid substitutions (AASs) to affect conserved sites. Apart from significant difference in pairwise alignment identity of candidate AASs between diploids and tetraploids (main text, Fig. 1D), we also calculated the difference between ancestral and derived amino acids in two other functional properties: hydrophobicity (contact angle hydrophobicity (Zhu et al. 2016)) and isoelectric point (pI), as

these metrics correlate with the predicted effect of the amino acid change on protein structure. Mean hydrophobicity difference candidate AASs identified among diploids was 24.5 and in tetraploids 39.3, showing an apparent increase in tetraploids; this difference was nearly but not quite statistically significant (p-value = 0.06, Wilcoxon rank sum test, Figure S1). Mean difference in pI for AASs identified among diploids was 1.59 and for ploidy-differentiated AASs 1.81. While this was also higher for tetraploids, the difference was not statistically significant (p-value = 0.18, Wilcoxon rank sum test, Figure S1). We further observed that meiosis proteins which are more strongly conserved (with higher alignment identity) during diploid plant evolution were less frequently used in *A. arenosa* tetraploid adaptation (Figure S5). This suggests that there are strong constraints to the evolution of some meiosis proteins, which are shared between diploid and tetraploid states and which prevent them from being used during rapid adaptation of tetraploids.

A very serious functional consequence of AASs is if a gene gains or loses a start or stop codon or has a different splice variant, thus, we also scanned all variant sites in meiotic genes for these high-effect variants, using SnpEff annotations (Cingolani et al. 2012). Across all meiosis genes, we identified only two candidate high-effect substitutions. One was a predicted splice site variant in ASY3, identified as a candidate in the tetraploids and the second was a premature stop codon in PDS5a found in the Baltic diploids.

We also identified a deletion in SCC3b, an interactor of REC8/SYN1 (Roig et al. 2014; Orgil et al. 2015), that leads to an early stop codon and truncates the last 26 amino acids from the protein. This version of the SCC3b was widespread in diploids but did not appear in the tetraploids. In fact, closer inspection suggested that the SCC3b was not functional in tetraploids, with an early stop codon in the second exon, possibly indicating that this loss of function mutation has been under selection in tetraploids.

Supplementary Text 3: Secondary protein structure prediction

We used prediction of secondary protein structures in PSIPRED (Buchan and Jones 2019) to ask whether candidate AASs in meiotic proteins are predicted to have consequences for the formation of coil (disordered), helix and sheet elements within these proteins.

The candidate tetraploid AASs in four candidate meiosis proteins with available structural information indeed lead to secondary protein structure variation. However, the candidate AASs and structural changes were not necessarily located in the same parts of sequence and the number of candidate AASs did not correlate with number of structure variations (Pearson's r = -0.33, p = 0.67, n = 4), which might be due to changes of local forces within the amino acid chain (Figure S2). The many amino acid polymorphisms at the ASY3 N-terminus, for instance, have only a minor impact on the secondary structure prediction, which is predicted to be disordered (Figure S2), meaning that local forces between amino

acid residues are low. Similar effects were found for REC8/SYN1 (Figure S2). In contrast, the C-terminus of ASY3, which is predicted to be highly structured, is more sensitive to changes in the amino acid sequence. In contrast, in ASY1, a relatively small number of amino acid polymorphisms that differ between diploids and tetraploids have a greater impact on the predicted secondary structure (Figure S2). Most of the predicted structural changes are located towards the C-terminus which includes a SWIRM domain postulated to be involved in chromatin binding and modification (Aravind and Iyer 2002). This gene is the most conserved among the meiosis proteins in the diploids, and also across the kingdoms (Grishaeva and Bogdanov 2014), and thus it is more likely that any change is functionally more important than in the proteins that show larger variation, such as ASY3.

Supplementary Text 4: Validation at the transcriptional level

We downloaded previously generated RNASeq data for diploid and tetraploid flower buds for SNO (W. Carpathian diploid), KZL (Pannonian diploid), and KAS (tetraploid) populations, 3 individuals each, from ENA for projects PRJEB34382 (ERP117274). The reads were mapped to *A. lyrata* reference using hisat2 (v 2.1.0) (Kim et al 2015).

Using the mapped RNASeq reads, we checked all candidate meiosis protein sequences for variability in splicing isoforms compared to the reference model. We identified irregularities in three candidate proteins (REC8/SYN1, PRD3 and PDS5b, Figure S6), potentially affecting five candidate amino acid substitutions (Arg353His in REC1/SYN1 and Pro306Thr, Asn292Ser, Glu277Gly, Thr269Lys and Thr260Ile in PRD3). While these could be due to the mapping issues or specific to the limited population sampling of RNASeq dataset, we cannot exclude that they represent real variations in splicing and thus that the effect of these five sites on meiosis protein evolution could be lower than we predicted. We further checked for splicing variation using SnpEff annotations of variants in our genomic dataset. Out of the 78 meiosis protein sequences, we identified only a single differentiated splicing variant, in ASY3.

To screen for possible gene expression levels differences between diploids and tetraploids, gene counts of the *A. lyrata*-mapped reads were produced using stringtie 1.3.5 (Pertea et al 2015). These count tables were uploaded to Degust (http://degust.erc.monash.edu/) and the count tables S13 were downloaded for contrasts SNO, KZL (W. Carpathian diploid and Pannonian diploid) and SNO, KAS (diploid and tetraploid) separately. These were intersected with the list of all 78 meiosis genes. We found out that all candidate meiosis genes were expressed in all three lineages, which makes our genetic variability-based predictions more likely to translate into the protein functions.

Supplemental References

Aravind L, Iyer LM. 2002. The SWIRM domain: a conserved module found in chromosomal proteins points to novel chromatin-modifying activities. Genome Biol. 3:research0039.1.

Bauer E, Falque M, Walter H, Bauland C, Camisan C, Campo L, Meyer N, Ranc N, Rincent R, Schipprack W, et al. 2013. Intraspecific variation of recombination rate in maize. Genome Biol. 14:1–17.

Bomblies K, Jones G, Franklin C, Zickler D, Kleckner N. 2016. The challenge of evolving stable polyploidy: could an increase in “crossover interference distance” play a central role? Chromosoma 125:287–300.

Bomblies K, Madlung A. 2014. Polyploidy in the Arabidopsis genus. Chromosom. Res. [Internet] 22:117–134. Available from: http://www.ncbi.nlm.nih.gov/pubmed/24788061

Buchan DWA, Jones DT. 2019. The PSIPRED Protein Analysis Workbench: 20 years on. Nucleic Acids Res. [Internet] 47. Available from: https://academic.oup.com/nar/article-abstract/47/W1/W402/5480136

Bulankova P, Riehs-Kearnan N, Nowack MK, Schnittger A, Riha K. 2010. Meiotic progression in Arabidopsis is governed by complex regulatory interactions between SMG7, TDM1, and the meiosis I-specific cyclin TAM. Plant Cell 22:3791–3803.

Cingolani P, Platts A, Wang LL, Coon M, Nguyen T, Wang L, Land SJ, Lu X, Ruden DM. 2012. A program for annotating and predicting the effects of single nucleotide polymorphisms, SnpEff: SNPs in the genome of Drosophila melanogaster strain w1118; iso-2; iso-3. Fly (Austin). [Internet] 6:80–92. Available from: http://www.ncbi.nlm.nih.gov/pubmed/22728672

Comai L. 2005. The advantages and disadvantages of being polyploid. Nat. Rev. Genet. 6:836–846.

Grishaeva TM, Bogdanov YF. 2014. Conservation and variability of synaptonemal complex proteins in phylogenesis of eukaryotes. Int. J. Evol. Biol. [Internet] 2014. Available from: http://www.ncbi.nlm.nih.gov/pubmed/25147749

Guiraldelli MF, Felberg A, Almeida LP, Parikh A, de Castro RO, Pezza RJ. 2018. SHOC1 is a ERCC4-(HhH)2-like protein, integral to the formation of crossover recombination intermediates during mammalian meiosis.Lichten M, editor. PLOS Genet. [Internet] 14:e1007381. Available from: http://dx.plos.org/10.1371/journal.pgen.1007381

Hollister JD, Arnold BJ, Svedin E, Xue KS, Dilkes BP, Bomblies K. 2012. Genetic Adaptation Associated with Genome-Doubling in Autotetraploid Arabidopsis arenosa.Mauricio R, editor. PLoS Genet. [Internet] 8:e1003093. Available from: http://dx.plos.org/10.1371/journal.pgen.1003093

Kim D, Langmead B, Salzberg SL. 2015. HISAT: a fasta spliced aligner with low memory requirements. Nature Methods. Available from: https://www.nature.com/articles/nmeth.3317

Johnston SE, Bérénos C, Slate J, Pemberton JM. 2016. Conserved Genetic Architecture Underlying Individual Recombination Rate Variation in a Wild Population of Soay Sheep (Ovis aries). Available from: www.genetics.org/cgi/data/genetics.

Lambing C, Franklin FCH, Wang C-JR. 2017. Understanding and Manipulating Meiotic Recombination in Plants. Plant Physiol. [Internet] 173:1530–1542. Available from: http://www.ncbi.nlm.nih.gov/pubmed/28108697

Macaisne N, Novatchkova M, Peirera L, Vezon D, Jolivet S, Froger N, Chelysheva L, Grelon M, Mercier R. 2008. SHOC1, an XPF Endonuclease-Related Protein, Is Essential for the Formation of Class I Meiotic Crossovers. Curr. Biol. [Internet] 18:1432–1437. Available from: http://www.ncbi.nlm.nih.gov/pubmed/18812090

Macaisne N, Vignard J, Mercier R. 2011. SHOC1 and PTD form an XPF–ERCC1-like complex that is required for formation of class I crossovers. J. Cell Sci. [Internet] 124. Available from: http://jcs.biologists.org/content/124/16/2687.short

Morgan C, Zhang H, Henry CE, Franklin FCH, Bomblies K. 2020. Derived alleles of two axis proteins affect meiotic traits in autotetraploid *Arabidopsis arenosa*. Proc. Natl. Acad. Sci. [Internet]:201919459. Available from: http://www.pnas.org/lookup/doi/10.1073/pnas.1919459117

Orgil O, Matityahu A, Eng T, Guacci V, Koshland D, Onn I. 2015. A Conserved Domain in the Scc3 Subunit of Cohesin Mediates the Interaction with Both Mcd1 and the Cohesin Loader Complex. PLoS Genet. 11.

Pertea et al. 2016. Transcript-level expression analysis of RNA-seq experiments with HISAT, StringTie and Ballgown. Nature Protocols. Available from: 10.1038/nprot.2016.095

Powell D. Degust: interactive RNA-seq analysis, DOI: 10.5281/zenodo.3258932

Pradillo M, Knoll A, Oliver C, Varas J, Corredor E, Puchta H, Santos JL. 2015. Involvement of the cohesin cofactor PDS5 (SPO76) during meiosis and DNA repair in Arabidopsis thaliana. Front. Plant Sci. 6.

Riehs N, Akimcheva S, Puizina J, Bulankova P, Idol RA, Siroky J, Schleiffer A, Schweizer D, Shippen DE, Riha K. 2008. Arabidopsis SMG7 protein is required for exit from meiosis. J. Cell Sci. [Internet] 121:2208–2216. Available from: http://www.ncbi.nlm.nih.gov/pubmed/18544632

Roig MB, Löwe J, Chan K-L, Beckouët F, Metson J, Nasmyth K. 2014. Structure and function of cohesin's Scc3/SA regulatory subunit. FEBS Lett. [Internet] 588:3692–3702. Available from: http://doi.wiley.com/10.1016/j.febslet.2014.08.015

Sandor C, Li W, Coppieters W, Druet T, Charlier C, Georges M. 2012. Genetic variants in REC8, RNF212, and PRDM9 influence male recombination in cattle. PLoS Genet. 8.

Stenberg P, Saura A. 2013. Meiosis and its deviations in polyploid animals. Cytogenet. Genome Res. [Internet] 140:185–203. Available from: http://www.ncbi.nlm.nih.gov/pubmed/23796636

Tessé S, Bourbon HM, Debuchy R, Budin K, Dubois E, Liangran Z, Antoine R, Piolot T, Kleckner N, Zickler D, et al. 2017. Asy2/Mer2: An evolutionarily conserved mediator of meiotic recombination, pairing, and global chromosome compaction. Genes Dev. 31:1880–1893.

Tong K, Skibbens R V. 2015. Pds5 regulators segregate cohesion and condensation pathways in Saccharomyces cerevisiae. Proc. Natl. Acad. Sci. U. S. A. 112:7021–7026.

Verver DE, Hwang GH, Jordan PW, Hamer G. 2016. Resolving complex chromosome structures during meiosis: versatile deployment of Smc5/6. Chromosoma [Internet] 125:15–27. Available from: http://www.ncbi.nlm.nih.gov/pubmed/25947290

Wells JN, Gligoris TG, Nasmyth KA, Marsh JA. 2017. Evolution of condensin and cohesin complexes driven by replacement of Kite by Hawk proteins. Curr. Biol. 27:R17–R18.

Wright KM, Arnold B, Xue K, Surinov M, O'connell J, Bomblies K, Wright S. 2015. Selection on Meiosis Genes in Diploid and Tetraploid Arabidopsis arenosa. Mol. Biol. Evol. [Internet] 32:944–955. Available from: https://academic.oup.com/mbe/article-abstract/32/4/944/1074938

Yang C, Hamamura Y, Sofroni K, Böwer F, Stolze SC, Nakagami H, Schnittger A. 2019. SWITCH 1/DYAD is a WINGS APART-LIKE antagonist that maintains sister chromatid cohesion in meiosis. Nat. Commun. 10:1–15.

Yant L, Hollister JD, Wright KM, Arnold BJ, Higgins JD, Franklin FCH, Bomblies K. 2013. Meiotic Adaptation to Genome Duplication in Arabidopsis arenosa. Available from: http://www.sciencedirect.com/science/article/pii/S0960982213011251

Zhu C, Gao Y, Li H, Meng S, Li L, Francisco JS, Zeng XC. 2016. Characterizing hydrophobicity of amino acid side chains in a protein environment via measuring contact angle of a water nanodroplet on planarpeptide network. Proc. Natl. Acad. Sci. U. S. A. 113:12946–12951.

Supplemental Figures

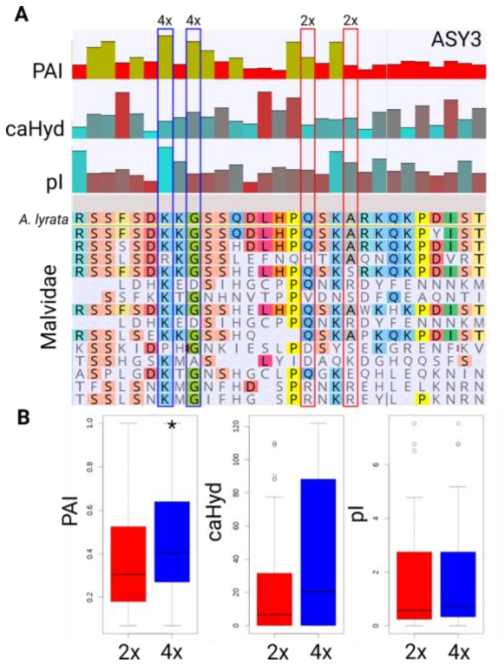

Figure S1. Level of evolutionary conservation of sites selected in diploids and tetraploids. A: An example of alignment from ASY3: diploid variants (red) in lower pairwise alignment identity (PAI) sites than tetraploid (blue). B: Boxplots of difference in PAI, caHydrophobicity and pI in sites selected in diploids and tetraploids. Asterisk is showing the significant difference (p-value = 0.002, Wilcoxon rank sum test).

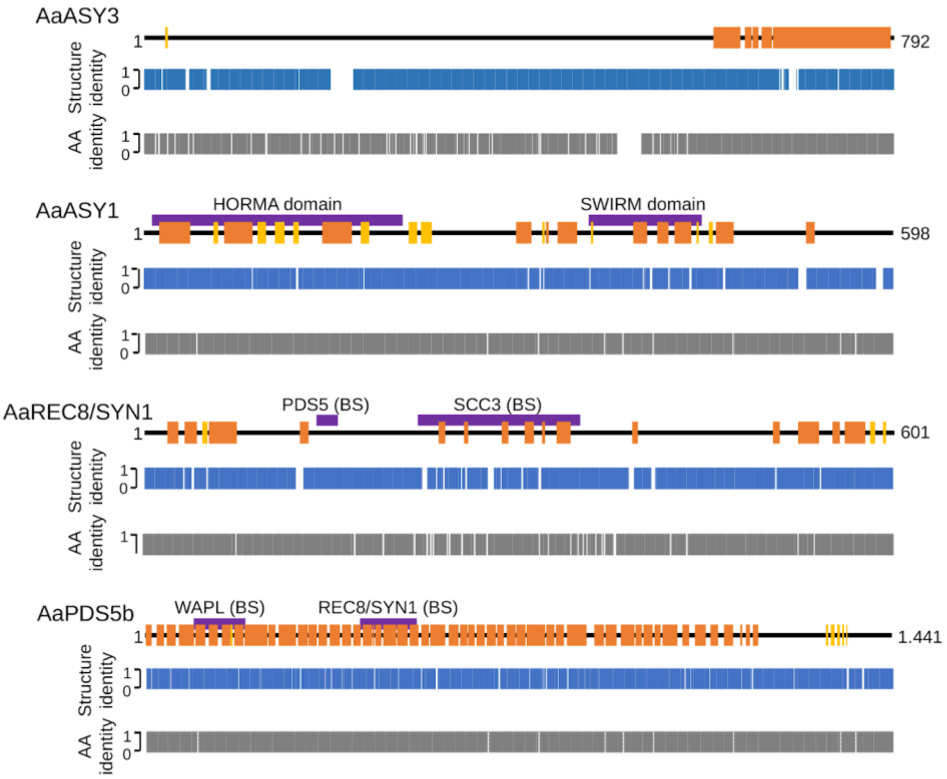

Figure S2. Candidate tetraploid amino acid substitutions lead to secondary protein structure variation. Shown are cartoons of secondary protein structures from diploid *A. arenosa* meiosis proteins (upper lane; in orange = helix elements, in yellow = sheet elements, and black line = disordered protein regions). The pairwise comparison of predicted secondary protein structures from sequences of diploid and tetraploid *A. arenosa* lineages (middle lane, Structure identity plots), and the identity of their amino acid sequences (lower lane, AA identity plots). Gaps are sites with zero identity. Protein binding sites and functional domains identified in other eukaryotes are shown above the secondary structure plot.

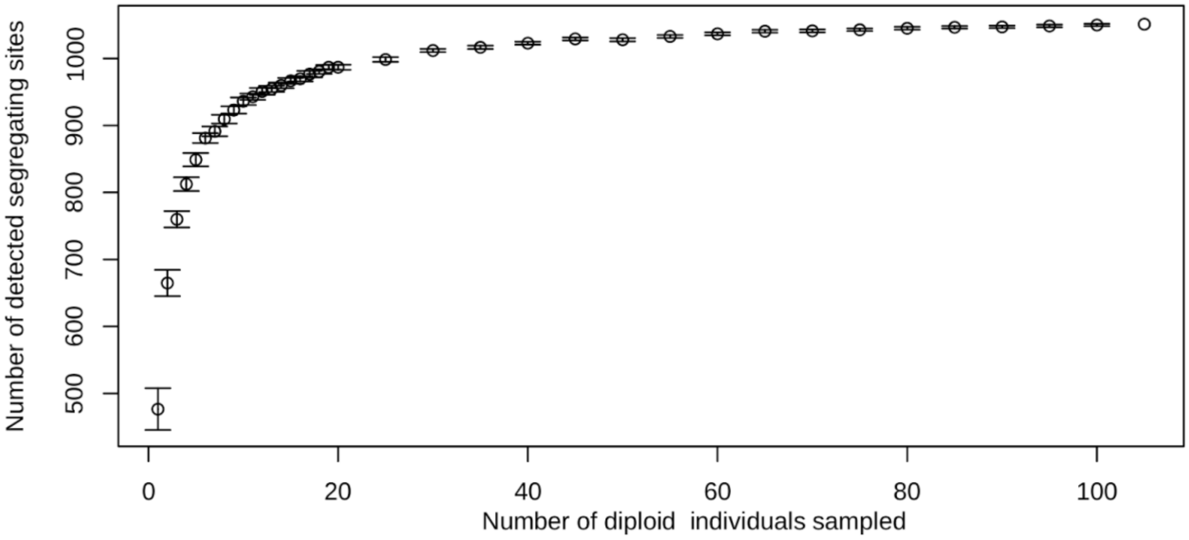

Figure S3: a rarefaction curve displaying the number of segregating sites detected based on the sampling intensity of *A. arenosa* diploid individuals across its full species range (fig. 1A). The shape of the curve implies that a sample of 105 individuals is sufficient to converge on the true diversity. We performed the analysis across the genome-wide set of 1269 candidate amino acid substitution differentiated between diploids and tetraploids, out of which 1064 were identified to segregate in the full set of 105 diploids. Confidence intervals were derived from 100 replicates of each subsampling.

A. PRD3

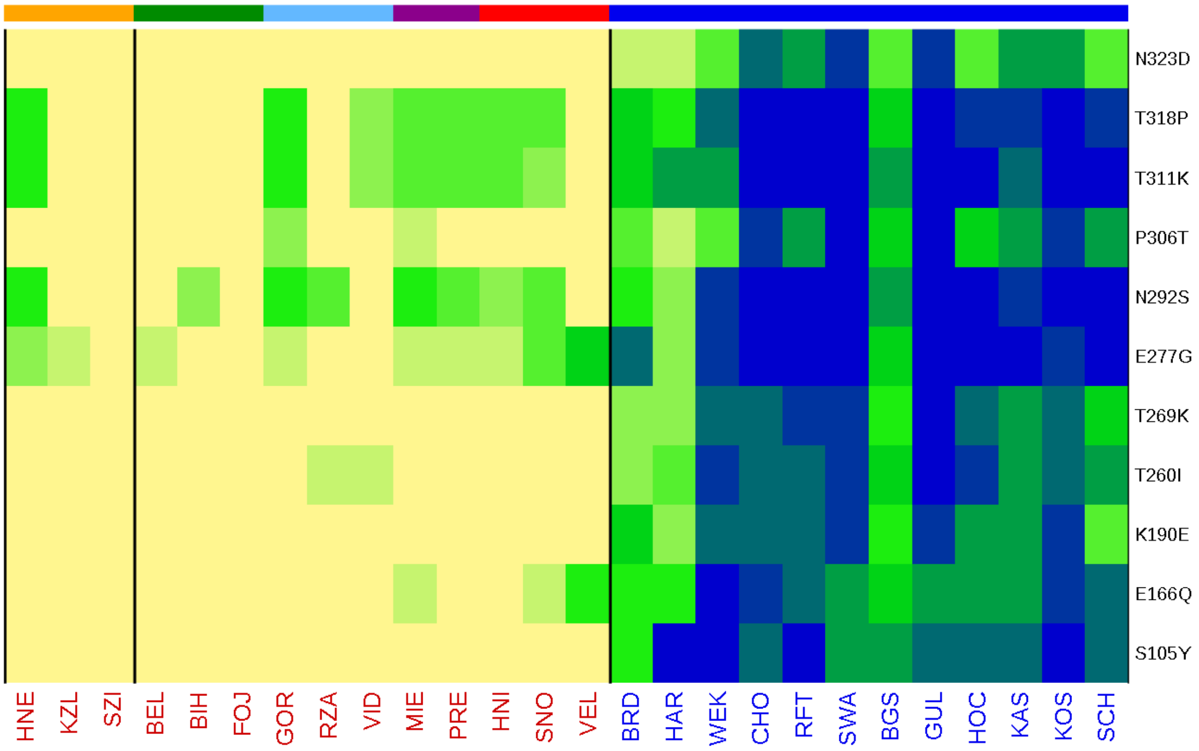

B. ZYP1a

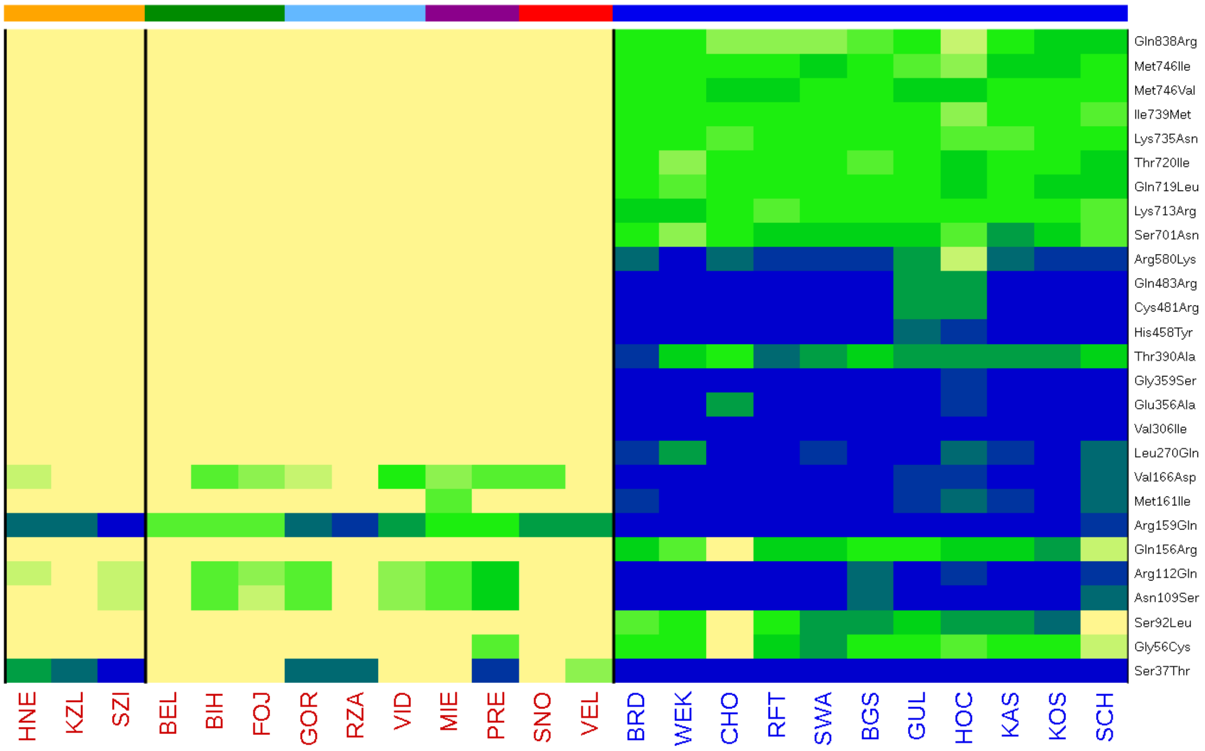

C. ZYP1b

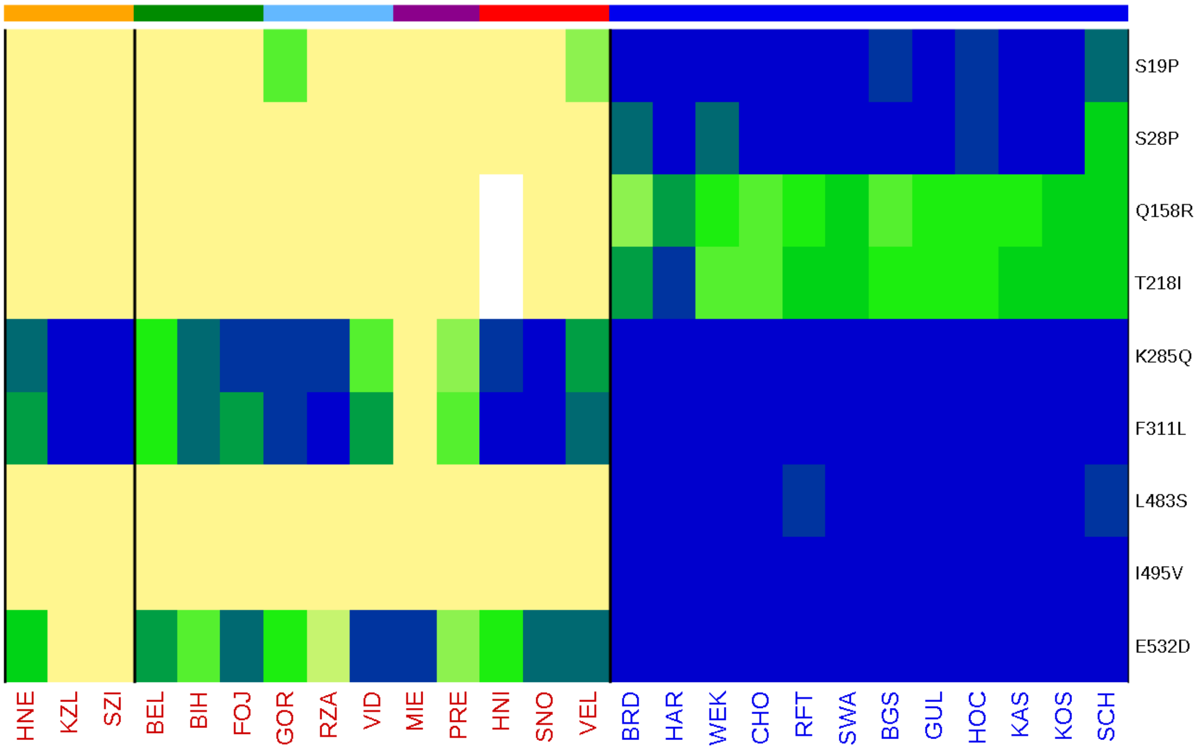

D. ASY1

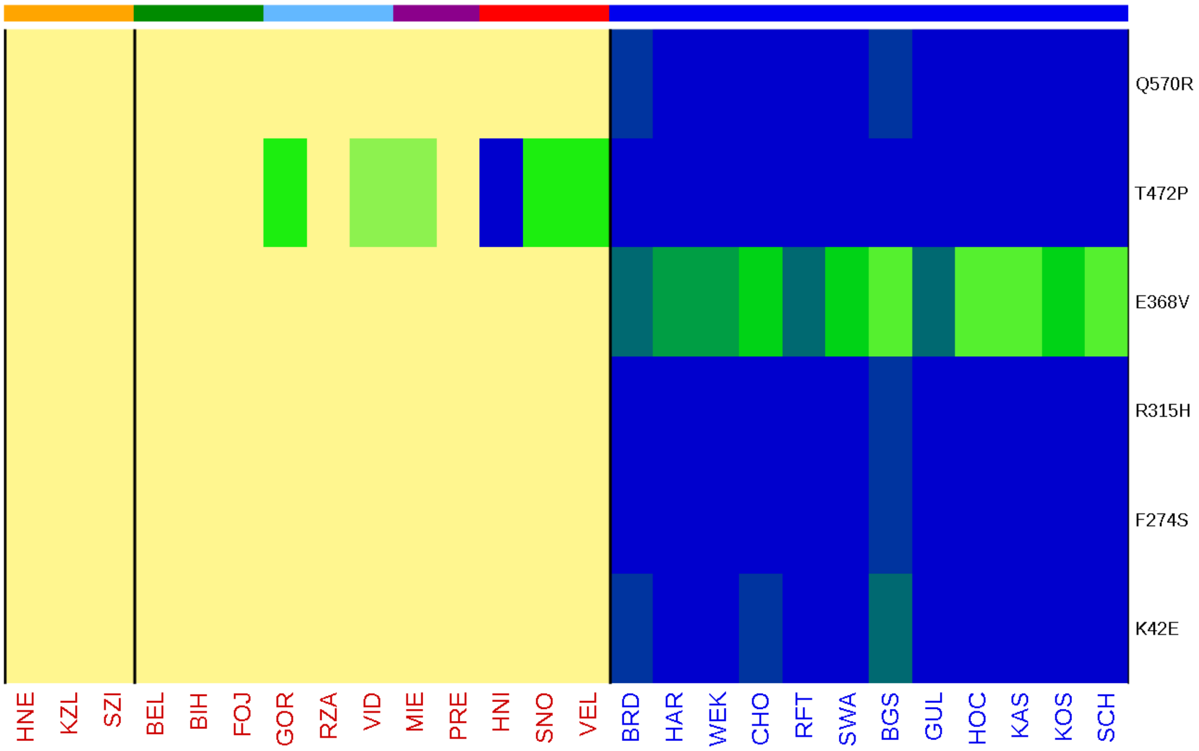

E. PDS5b

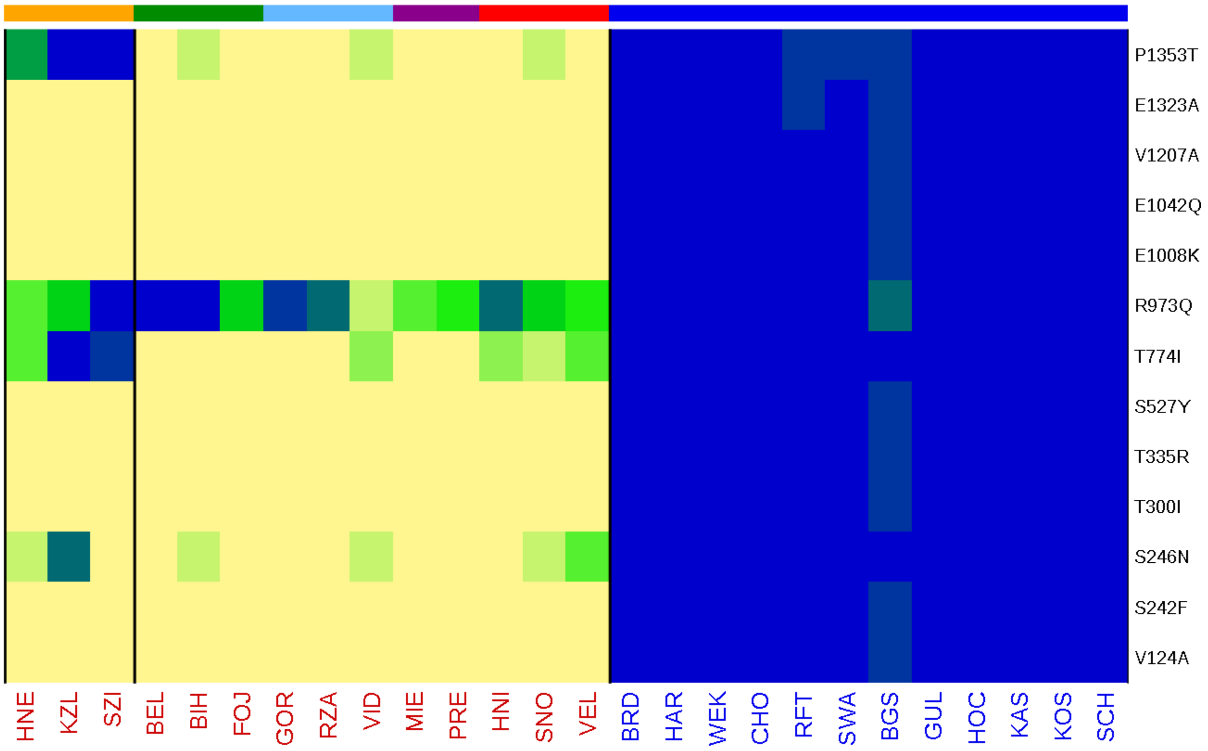

F. ASY3

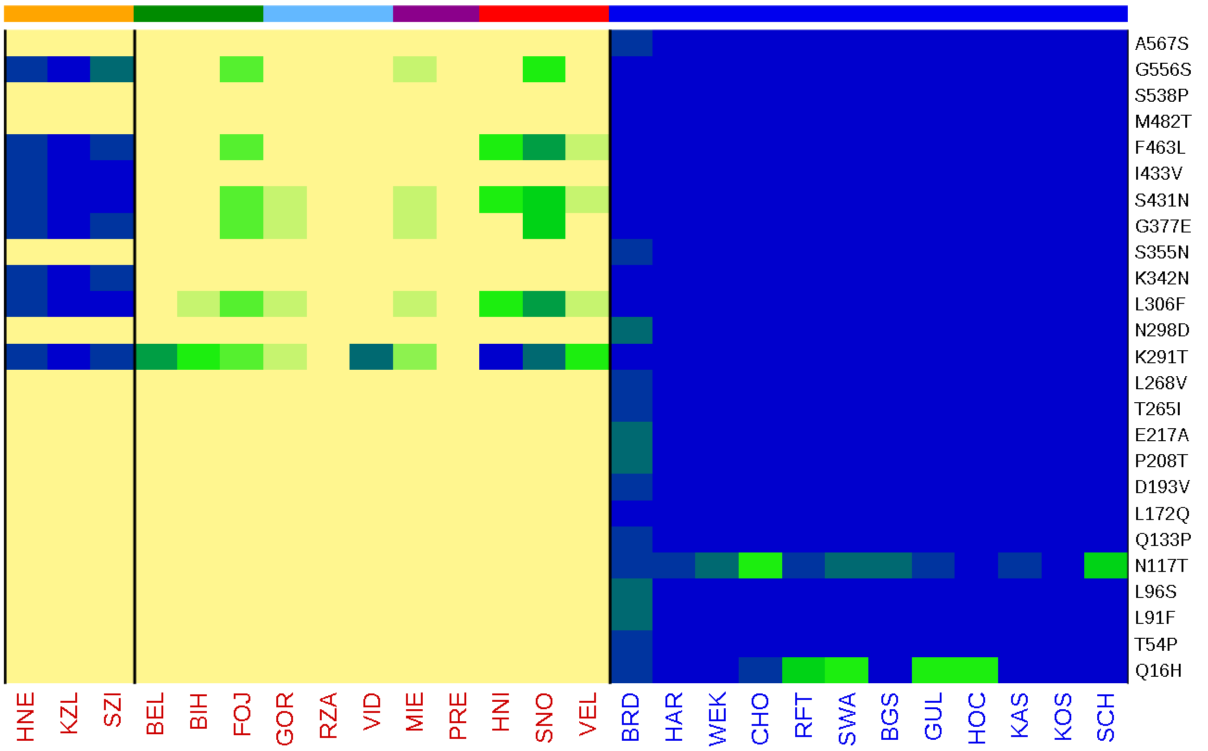

G. REC8/SYN1

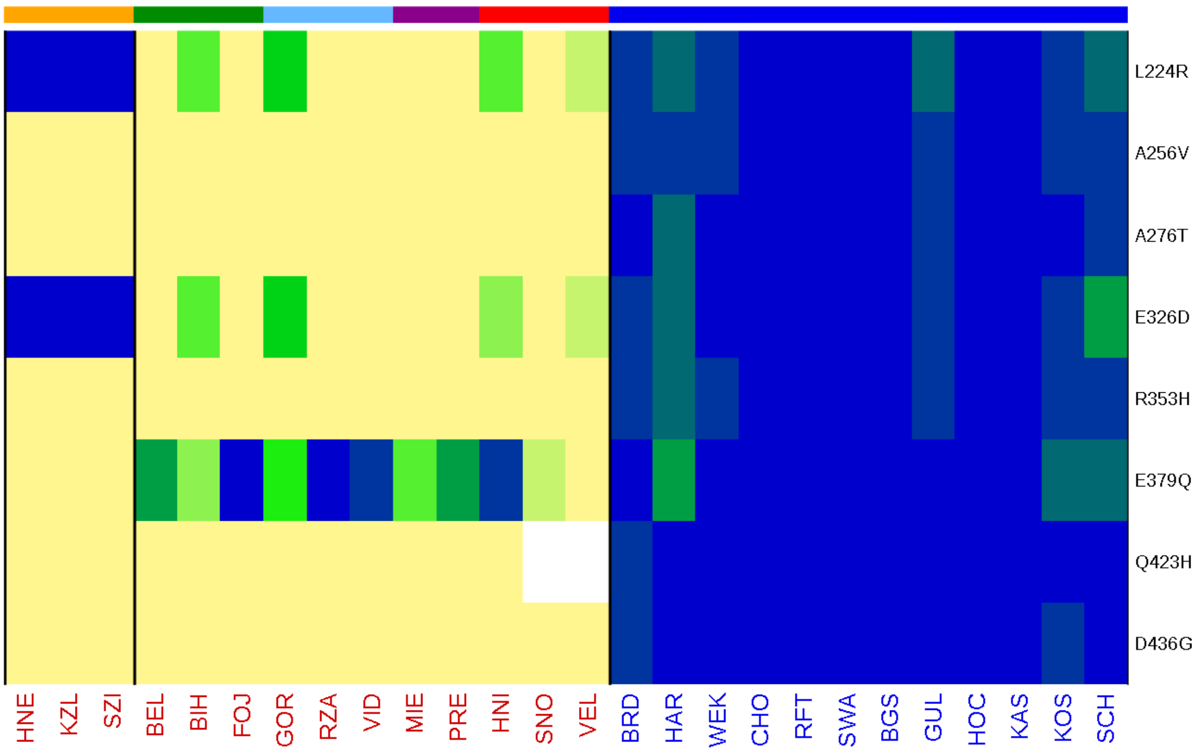

H. SMG7

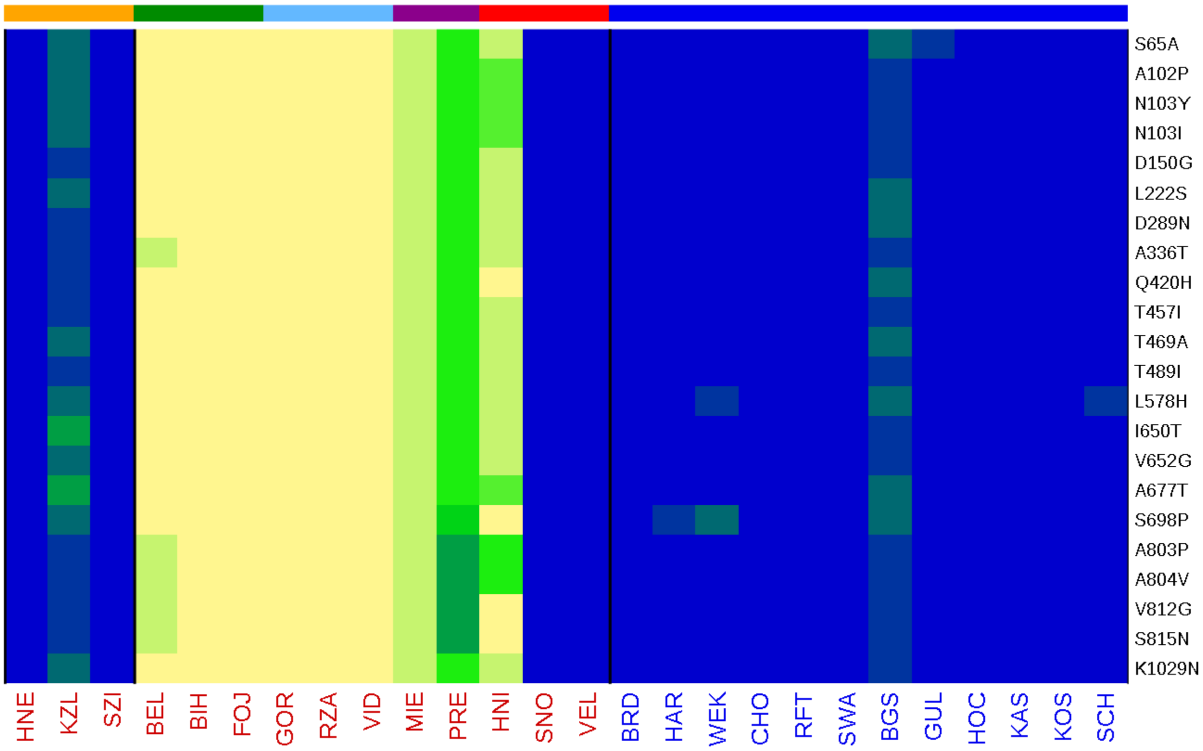

I. DYAD

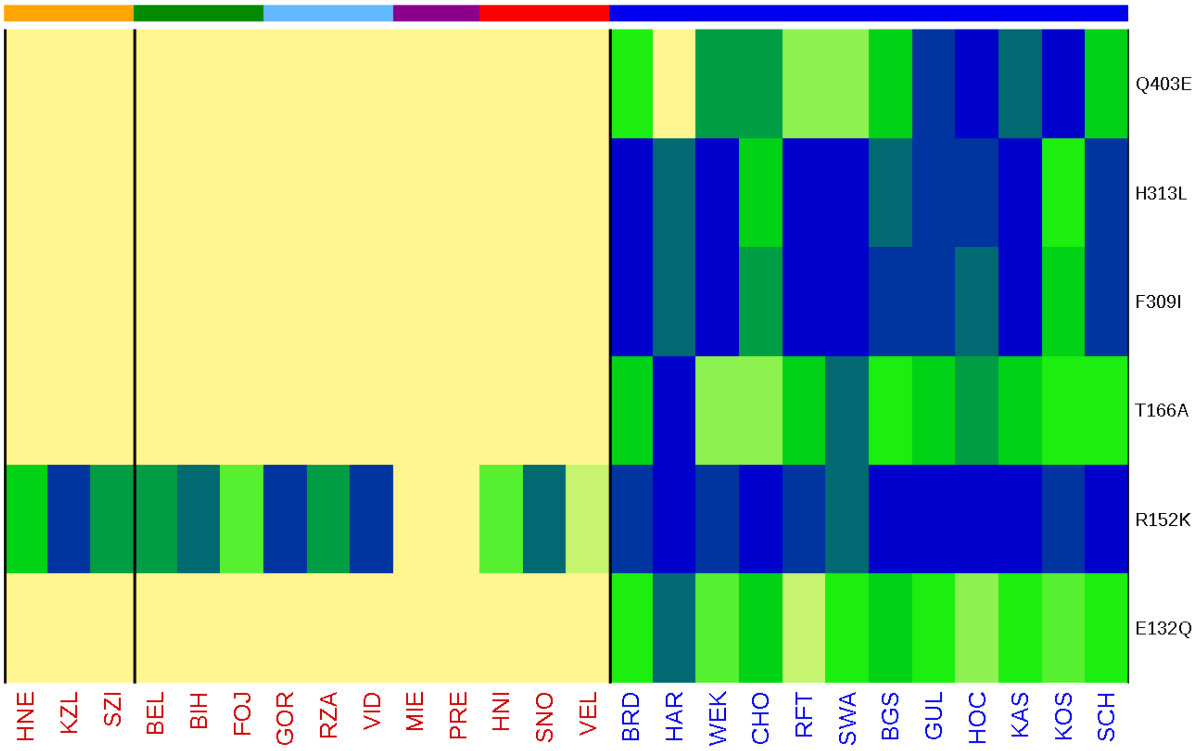
Q403E
H313L
F309I
T166A
R152K
E132Q
HNE KZL SZI BEL BIH FOJ GOR RZA VID MIE PRE HNI SNO VEL BRD HAR WEK CHO RFT SWA BGS GUL HOC KAS KOS SCH

J. SCC4

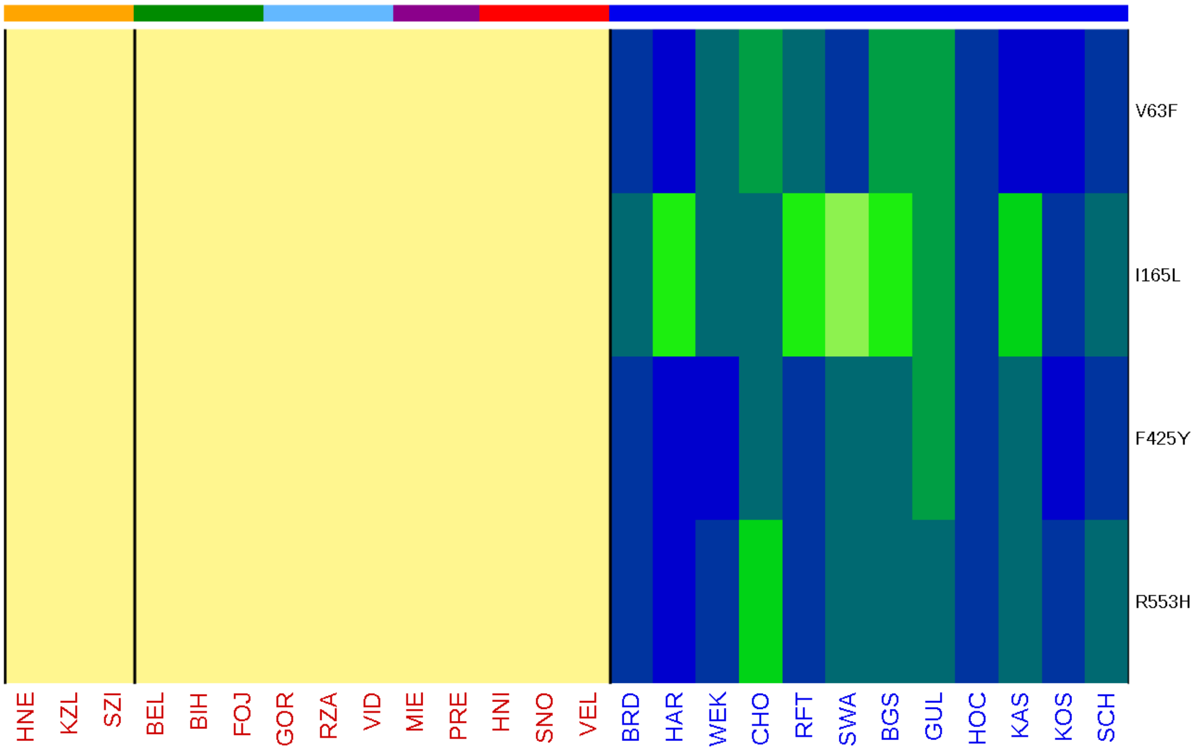
V63F
I165L
F425Y
R553H
HNE KZL SZI BEL BIH FOJ GOR RZA VID MIE PRE HNI SNO VEL BRD HAR WEK CHO RFT SWA BGS GUL HOC KAS KOS SCH

K. SHOC1

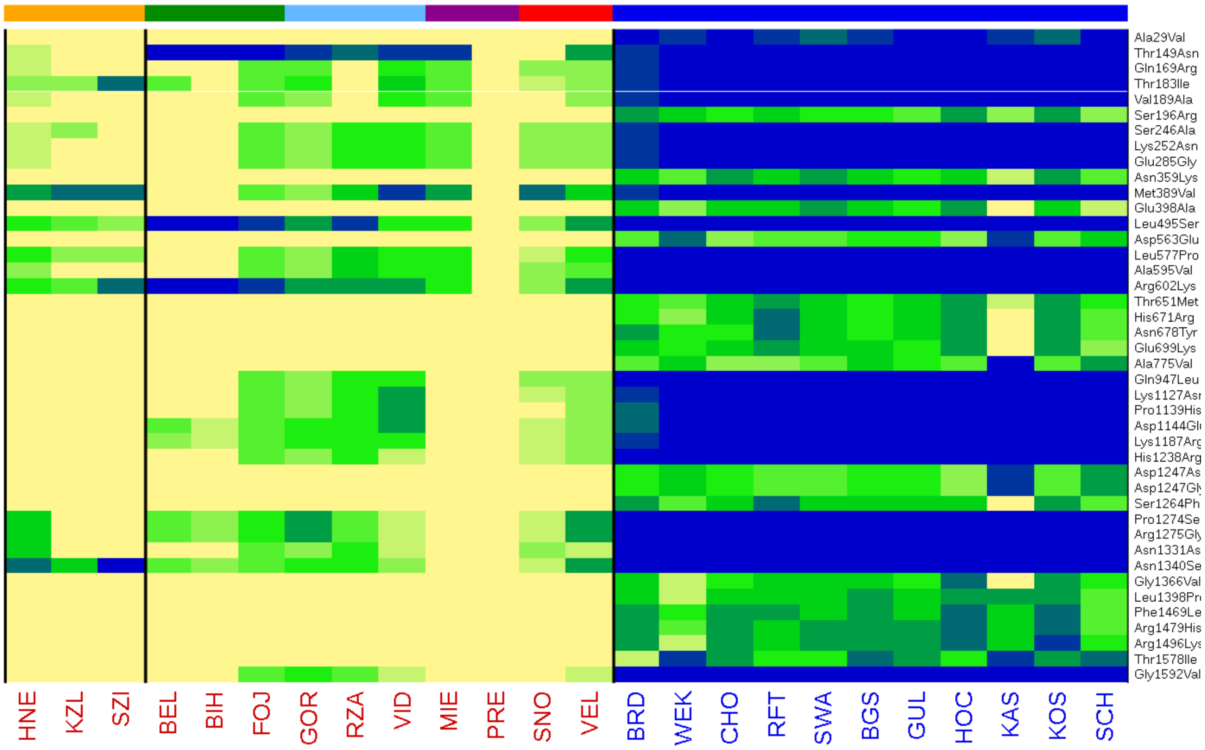

Figure S4. Allele frequencies (AF) of amino acid substitution (AAS) in candidate meiosis proteins demonstrating limited standing variation in diploids. A-K: Upper bar: *A. arenosa* lineages (color coded based on fig. 1A; orange: Pannonian diploids, green: Dinaric diploids, light blue: S. Carpathian diploids, magenta: Baltic diploids, red: W. Carpathian diploids, blue tetraploids), heatmap colors: yellow: AF = 0 (fixed reference-like allele), green: 0 < AF < 1, blue: AF = 1 (fixed alternative allele), white: no data available. X-axis labels: population codes of *A. arenosa* populations (Table S1). Red text: diploids, blue text: tetraploids. Y-axis labels: amino acid substitutions ranked by their position in the protein.

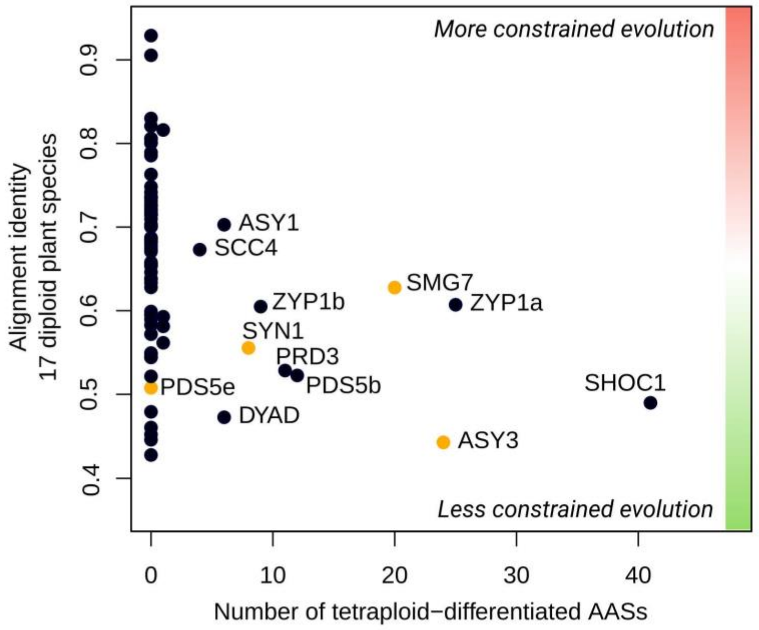

Figure S5: The level of evolutionary constraints acting on diploid meiosis protein sequences and the differentiation of meiosis proteins in tetraploids. X-axis: Number of candidate tetraploid-differentiated amino acid substitutions identified in this study, for the 78 meiosis proteins studied here. Value of zero means no signature of positive selection. Y-axis: alignment identity (a proxy for evolutionary constraints acting on the protein sequence) across orthologs of 17 diploid species related to *A. arenosa*. Dots with labels represent proteins showing signatures of selection in the Pannonian diploids and/or tetraploids. Orange dots show those selected (also) in the Pannonian lineage.

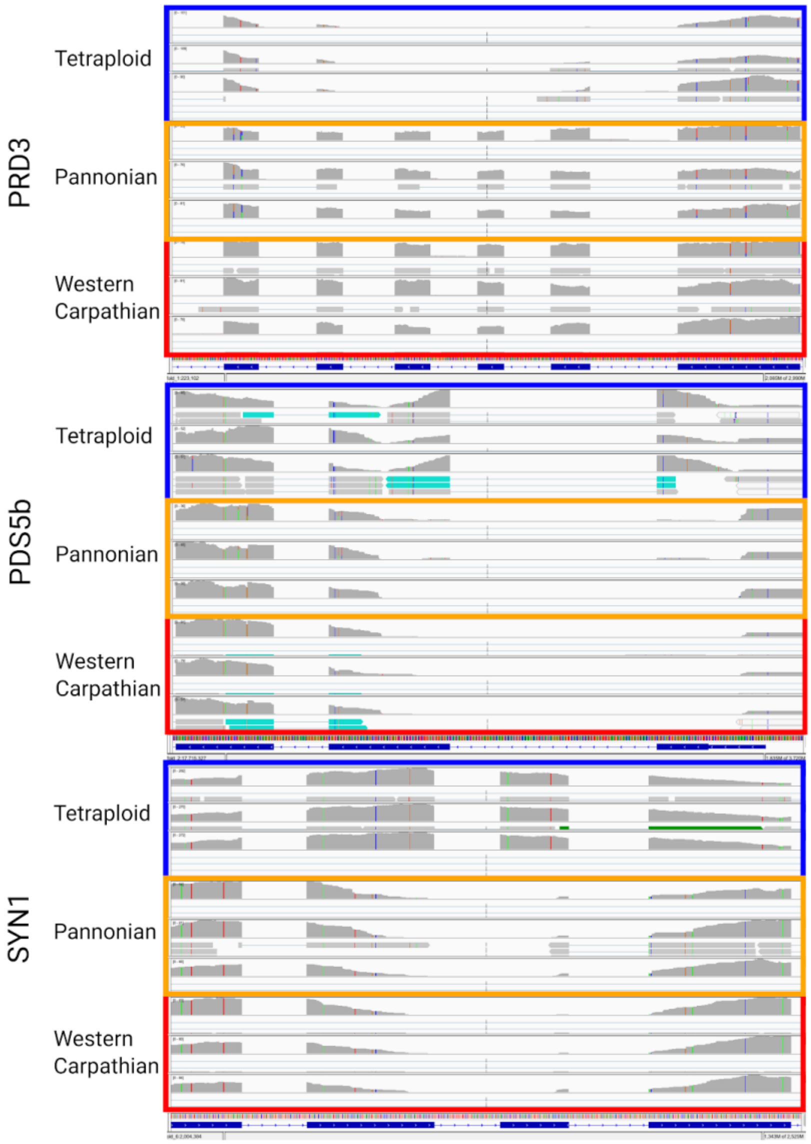

Figure S6: The reported irregularities in RNASeq reads mapping among populations in three candidate proteins, which could mirror variability in splicing isoforms between ploidies. Blue boxes with arrows below each sequence alignment represent exons. Regions of missing mapping overlap with five candidate AASs in PRD3, none in PDS5b and one in REC8/SYN1.

Supplemental Tables

Table S1: The full set of 291 range-wide individual sequences of A. arenosa (from Monnahan et al., 2019) which we subsampled to the datasets used here. Individual: individual code as used in Monnahan et al., 2019, Lin: genetic lineage (from Monnahan et al., 2019), Sub: dataset with balanced sample sizes across lineages and ploidies, used here for most of the analyses, SV: dataset including full set of diploid individuals, used to search for standing variation in diploids. 1 - included in the dataset, 0 - excluded from the dataset after the subsampling.

Individual	Lin	Sub	SV	Individual	Lin	Sub	SV
BEL_001_c	Dinaric	0	1	PRE_001_o	Baltic	1	1
BEL_001_f	Dinaric	0	1	PRE_001_p	Baltic	1	1
BEL_001_g	Dinaric	1	1	PRE_001_q	Baltic	1	1
BEL_001_j	Dinaric	0	1	RFT_002_1	Tetraploids	0	0
BEL_001_l	Dinaric	1	1	RFT_003_1	Tetraploids	1	1
BEL_001_m	Dinaric	1	1	RFT_006_1	Tetraploids	1	1
BEL_001_n	Dinaric	1	1	RFT_008_1	Tetraploids	0	0
BEL_001_o	Dinaric	1	1	RFT_009_1	Tetraploids	1	1
BGS_001_1	Tetraploids	0	0	RFT_010_1	Tetraploids	0	0
BGS_003_1	Tetraploids	0	0	RFT_011_1	Tetraploids	0	0
BGS_004_1	Tetraploids	0	0	RFT_012_1	Tetraploids	1	1
BGS_007_1	Tetraploids	0	0	RFT_016_1	Tetraploids	0	0
BGS_009_1	Tetraploids	0	0	RFT_017_1	Tetraploids	0	0
BGS_020_1	Tetraploids	0	0	RFT_023_1	Tetraploids	1	1
BGS_021_1	Tetraploids	0	0	RFT_024_1	Tetraploids	0	0
BGS_022_1	Tetraploids	0	0	RZA_008_1	Scarp.	0	1
BIH_001_a	Dinaric	0	1	RZA_010_1	Scarp.	1	1
BIH_001_e	Dinaric	0	1	RZA_013_1	Scarp.	1	1
BIH_001_f	Dinaric	1	1	RZA_017_1	Scarp.	0	1
BIH_001_h	Dinaric	0	1	RZA_018_1	Scarp.	1	1
BIH_001_j	Dinaric	1	1	RZA_019_1	Scarp.	0	1
BIH_001_k	Dinaric	1	1	RZA_019_2	Scarp.	0	1
BIH_001_l	Dinaric	0	1	RZA_024_1	Scarp.	0	1
BIH_001_p	Dinaric	1	1	RZA_025_1	Scarp.	0	1
BRD_001_a	Tetraploids	1	1	SCH_001_a	Tetraploids	0	0
BRD_001_c	Tetraploids	1	1	SCH_001_b	Tetraploids	0	0
BRD_001_d	Tetraploids	1	1	SCH_001_c	Tetraploids	0	0
BRD_001_i	Tetraploids	1	1	SCH_001_d	Tetraploids	0	0
BRD_001_m	Tetraploids	1	1	SCH_001_f	Tetraploids	0	0
CHO_001_a	Tetraploids	0	0	SCH_001_g	Tetraploids	0	0
CHO_001_b	Tetraploids	0	0	SCH_001_h	Tetraploids	0	0
CHO_001_c	Tetraploids	1	1	SNO_003_1	Wcarp.	1	1
CHO_001_d	Tetraploids	0	0	SNO_004_1	Wcarp.	1	1

CHO_001_e	Tetraploids	0	0	SNO_005_1	Wcarp.	1	1
CHO_001_f	Tetraploids	0	0	SNO_009_1	Wcarp.	0	1
CHO_001_g	Tetraploids	0	0	SNO_023_1	Wcarp.	0	1
CHO_001_h	Tetraploids	0	0	SPI_002_1	Tetraploids	0	0
CRO_001_bb	A. croatica	0	0	SPI_004_1	Tetraploids	0	0
CRO_001_be	A. croatica	0	0	SPI_005_1	Tetraploids	0	0
CRO_002_b	A. croatica	0	0	SPI_014_1	Tetraploids	0	0
CRO_002_g	A. croatica	0	0	SPI_016_1	Tetraploids	0	0
DFS_001_1	Tetraploids	0	0	SPI_022_1	Tetraploids	0	0
DRA_001_b	Tetraploids	0	0	SPI_025_1	Tetraploids	0	0
DRA_001_c	Tetraploids	0	0	SPI_026_1	Tetraploids	0	0
DRA_001_e	Tetraploids	0	0	SPI_027_1	Tetraploids	0	0
DRA_001_h	Tetraploids	0	0	SPI_029_1	Tetraploids	0	0
DRA_001_i	Tetraploids	0	0	SPI_030_1	Tetraploids	0	0
DRA_001_j	Tetraploids	0	0	SPI_031_1	Tetraploids	0	0
DRA_001_l	Tetraploids	0	0	SPI_034_1	Tetraploids	0	0
DRA_001_o	Tetraploids	0	0	STE_002_1	Tetraploids	0	0
FOJ_001_b	Dinaric	1	1	STE_005_1	Tetraploids	0	0
FOJ_001_c	Dinaric	1	1	STE_006_1	Tetraploids	0	0
FOJ_001_d	Dinaric	1	1	STE_008_1	Tetraploids	0	0
FOJ_001_e	Dinaric	1	1	STE_010_1	Tetraploids	0	0
FOJ_001_f	Dinaric	1	1	STE_013_1	Tetraploids	0	0
FOJ_001_g	Dinaric	1	1	STE_014_1	Tetraploids	0	0
FOJ_001_h	Dinaric	1	1	STE_047_1	Tetraploids	0	0
FOJ_001_n	Dinaric	0	1	SWA_001_1	Tetraploids	1	1
GOR_001_a	Scarp.	1	1	SWA_002_1	Tetraploids	1	1
GOR_001_b	Scarp.	1	1	SWA_003_1	Tetraploids	1	1
GOR_001_c	Scarp.	1	1	SWA_004_1	Tetraploids	1	1
GOR_001_e	Scarp.	1	1	SWA_005_1	Tetraploids	0	0
GOR_001_f	Scarp.	1	1	SWA_006_1	Tetraploids	0	0
GOR_001_p	Scarp.	1	1	SWA_007_1	Tetraploids	1	1
GOR_001_q	Scarp.	1	1	SWA_008_1	Tetraploids	0	0
GOR_001_t	Scarp.	1	1	SWA_009_1	Tetraploids	0	0
GUL_001_1	Tetraploids	1	1	SWA_010_1	Tetraploids	0	0
GUL_002_1	Tetraploids	1	1	SZI_003_1	Pannonian	0	1
GUL_003_1	Tetraploids	0	0	SZI_008_1	Pannonian	1	1
GUL_004_1	Tetraploids	0	0	SZI_012_1	Pannonian	1	1
GUL_005_1	Tetraploids	1	1	SZI_027_1	Pannonian	1	1
GUL_006_1	Tetraploids	1	1	SZI_029_1	Pannonian	1	1
GUL_007_1	Tetraploids	1	1	TBG_001_1	Tetraploids	0	0
GUL_008_1	Tetraploids	1	1	TBG_004_1	Tetraploids	0	0
GUL_009_1	Tetraploids	1	1	TBG_017_1	Tetraploids	0	0
GUL_010_1	Tetraploids	1	1	TBG_021_1	Tetraploids	0	0
HAR_005_1	Tetraploids	0	0	TBG_022_1	Tetraploids	0	0
HAR_006_1	Tetraploids	0	0	TKO_001_a	Tetraploids	0	0
HNE_002_1	Pannonian	1	1	TKO_001_h	Tetraploids	0	0

HNE_005_1	Pannonian	1	1	TKO_001_m	Tetraploids	0	0
HNE_011_1	Pannonian	1	1	TKO_001_n	Tetraploids	0	0
HNE_012_1	Pannonian	1	1	TKO_001_o	Tetraploids	0	0
HNE_014_1	Pannonian	1	1	TKO_001_p	Tetraploids	0	0
HNE_018_1	Pannonian	1	1	TKO_001_q	Tetraploids	0	0
HNE_019_1	Pannonian	1	1	TKO_001_t	Tetraploids	0	0
HNI_001_1	Wcarp.	0	1	TRD_001_a	Wcarp.	0	1
HNI_002_1	Wcarp.	1	1	TRD_001_ba	Wcarp.	0	1
HNI_003_1	Wcarp.	0	1	TRD_001_bs	Wcarp.	1	1
HNI_004_1	Wcarp.	0	1	TRD_001_m	Wcarp.	1	1
HOC_004_1	Tetraploids	1	1	TRD_001_n	Wcarp.	1	1
HOC_007_1	Tetraploids	0	0	TRD_001_p	Wcarp.	1	1
HOC_009_1	Tetraploids	0	0	TRE_004_1	Tetraploids	0	0
HOC_010_1	Tetraploids	1	1	TRE_006_1	Tetraploids	0	0
HOC_015_1	Tetraploids	1	1	TRE_010_1	Tetraploids	0	0
HOC_017_1	Tetraploids	1	1	TRE_011_1	Tetraploids	0	0
HOC_020_1	Tetraploids	1	1	TRE_013_1	Tetraploids	0	0
HOC_021_1	Tetraploids	1	1	TRE_014_1	Tetraploids	0	0
KAS_016_1	Tetraploids	1	1	TRE_021_1	Tetraploids	0	0
KAS_018_1	Tetraploids	1	1	TRE_022_1	Tetraploids	0	0
KAS_019_1	Tetraploids	1	1	TRT_001_bd	Tetraploids	0	0
KAS_027_1	Tetraploids	0	0	TRT_001_bk	Tetraploids	0	0
KAS_029_1	Tetraploids	1	1	TRT_001_co	Tetraploids	0	0
KAS_030_1	Tetraploids	1	1	TRT_001_dj	Tetraploids	0	0
KAS_031_1	Tetraploids	1	1	TRT_001_u	Tetraploids	0	0
KAS_032_1	Tetraploids	1	1	TRT_001_v	Tetraploids	0	0
KOS_001_1	Tetraploids	0	0	TRT_001_w	Tetraploids	0	0
KOS_005_1	Tetraploids	0	0	TRT_001_x	Tetraploids	0	0
KOS_007_1	Tetraploids	1	1	TZI_001_1	Tetraploids	0	0
KOS_008_1	Tetraploids	0	0	TZI_003_1	Tetraploids	0	0
KOS_010_1	Tetraploids	0	0	TZI_007_1	Tetraploids	0	0
KOS_011_1	Tetraploids	1	1	TZI_008_1	Tetraploids	0	0
KOS_012_1	Tetraploids	1	1	TZI_013_1	Tetraploids	0	0
KOW_001_a04	Tetraploids	0	0	TZI_018_1	Tetraploids	0	0
KOW_001_a05	Tetraploids	0	0	TZI_021_1	Tetraploids	0	0
KOW_001_a06	Tetraploids	0	0	TZI_027_1	Tetraploids	0	0
KOW_001_a07	Tetraploids	0	0	TZI_031_1	Tetraploids	0	0
KOW_001_a08	Tetraploids	0	0	VEL_001_a	Wcarp.	1	1
KOW_001_a09	Tetraploids	0	0	VEL_001_b	Wcarp.	1	1
KOW_001_a11	Tetraploids	0	0	VEL_001_d	Wcarp.	1	1
KOW_001_a12	Tetraploids	0	0	VEL_001_e	Wcarp.	1	1
KZL_004_1	Pannonian	1	1	VEL_001_k	Wcarp.	1	1
KZL_009_1	Pannonian	1	1	VEL_001_l	Wcarp.	1	1
KZL_017_1	Pannonian	1	1	VEL_001_n	Wcarp.	1	1
KZL_020_1	Pannonian	1	1	VEL_001_o	Wcarp.	1	1
KZL_021_1	Pannonian	1	1	VID_001_a	Scarp.	0	1

LAC_001_c	Tetraploids	0	0	VID_001_b	Scarp.	1	1
LAC_001_d	Tetraploids	0	0	VID_001_c	Scarp.	0	1
LAC_001_f	Tetraploids	0	0	VID_001_e	Scarp.	0	1
LAC_001_i	Tetraploids	0	0	VID_001_k	Scarp.	1	1
LAC_001_l	Tetraploids	0	0	VID_001_m	Scarp.	1	1
LAC_001_p	Tetraploids	0	0	VID_001_n	Scarp.	1	1
LAC_001_r	Tetraploids	0	0	VID_001_o	Scarp.	1	1
LAC_001_t	Tetraploids	0	0	WEK_001_a	Tetraploids	0	0
MIE_001_a	Baltic	1	1	WEK_001_b	Tetraploids	0	0
MIE_001_b	Baltic	1	1	WEK_001_c	Tetraploids	0	0
MIE_001_c	Baltic	1	1	WEK_001_d	Tetraploids	0	0
MIE_001_d	Baltic	1	1	WEK_001_e	Tetraploids	0	0
MIE_001_e	Baltic	1	1	WEK_001_f	Tetraploids	0	0
MIE_001_f	Baltic	1	1	WEK_001_h	Tetraploids	0	0
MIE_001_g	Baltic	1	1	WEK_001_i	Tetraploids	0	0
MIE_001_h	Baltic	1	1	ZAP_002_a03	Tetraploids	0	0
PRE_001_f	Baltic	1	1	ZAP_002_a05	Tetraploids	0	0
PRE_001_k	Baltic	1	1	ZAP_002_a06	Tetraploids	0	0
PRE_001_l	Baltic	1	1	ZAP_002_a07	Tetraploids	0	0
PRE_001_m	Baltic	1	1	ZAP_004_a11	Tetraploids	0	0
PRE_001_n	Baltic	1	1				

Table S2: Quality checks of the two novel draft reference assemblies

		Input sample QC	Final library QC		Assembly QC	
Sample	Ploidy	Input QuBit concentration (ng/ul)	Total input in GEM (ng)	QuBit concentration (ng/ul)	scaffold N50	cover of BUSCO plant specific genes
TBG	4x	0.403	0.504	1.01	33.9 kb	97.5 %
SNO	2x	0.516	0.645	2.86	2.2 Mb	98.5 %

Table S3: The list of 78 meiosis proteins analyzed here. ID: *A. lyrata* version 2.0 reference ID for the gene and protein. AA: length of the protein as the number of amino acids (including start and stop codons).

Protein	ID	Length (AA)	Protein	ID	Length (AA)
PRD3	AL1G10680	451	ATM	AL5G27530	2938
JASON	AL1G16670	487	TTN8	AL5G35750	1219
PHS1	AL1G21590	349	INO80	AL5G38840	1508
RECQ4A	AL1G21910	1190	SYN3	AL5G41480	697
WAPL1	AL1G22040	874	BRCA2B	AL6G10420	1152
SDS	AL1G26770	582	SGO2	AL6G13940	462
PDS5E	AL1G28100	1011	SYN1	AL6G15380	602
ZYP1A	AL1G35725	871	SMC6A	AL6G18000	781
ZYP1B	AL1G35730	877	PANS2	AL6G23160	197
FANCM	AL1G50380	1351	SCC2	AL6G26400	1848
HEI10	AL1G62040	305	SMC5	AL6G26800	1053
SPO11-2	AL2G11120	384	SCC1	AL6G27160	1031
WAPL2	AL2G15410	843	MCC1	AL6G27820	271
RECQ4B	AL2G15560	1154	SMG7	AL6G30890	1053
MMD1	AL2G23810	706	MLH1	AL6G42870	728
ASY1	AL2G25920	600	PMS1	AL6G50600	924
MEI1	AL2G37520	975	MLH3	AL7G15650	1181
PDS5B	AL2G37810	1423	PDS5C	AL7G20060	868
MHF2	AL2G39190	105	MUS81	AL7G21230	660
PDS5D	AL2G40580	832	MND1	AL7G23270	231
NBS1	AL3G12070	538	ESP	AL7G30550	2184
RAD9	AL3G16080	440	MSH4	AL7G37330	793
MCM8	AL3G20870	803	CAP-D3	AL7G39380	1316
SGO1	AL3G21800	572	PRD1	AL7G42200	1340
SPO11-1	AL3G24910	363	G-H2AX	AL7G50990	143
PANS1	AL3G26440	194	PDS5A	AL8G10260	1610
RAD54	AL3G32590	911	ZIP4	AL8G21200	938
MSH5	AL3G34200	794	XRI1	AL8G21800	302
DMC1	AL3G37220	345	MHF1	AL8G25120	185
CDC45	AL3G40790	601	DYAD	AL8G25590	588
RPA1A	AL3G43800	643	SCC4	AL8G25600	726
EME1A	AL4G11110	508	SHOC1	AL8G26680	1606
EME1B	AL4G11490	550	MRE11	AL8G29590	699
SMC3	AL4G20920	1202	SMC6B	AL8G38150	963
RAD50	AL4G27320	1317	BRCA2A	AL8G38500	909
RAD51C	AL4G44380	347	SMC2-1	AL8G39240	1176
ASY3	AL4G46460	793	RMI1	AL8G40620	641
SCC3	AL4G47570	1086	TOP3A	AL8G41160	924
MER3	AL5G17170	1132	RAD17	AL8G45460	589

Table S4: Within population metrics for each lineage in the subsampled dataset calculated for “neutral” four-fold degenerate SNPs genome-wide.

	num_alleles	num_indivs	num_snps	num_sites	nucl. diversity	Tajima’s D
Pannonian	16	8	257164	2810305	0.028	0.08
Dinaric	16	8	292968	3172710	0.029	0.17
S. Carp.	16	8	336045	3177770	0.032	0.03
Baltic	16	8	285914	3196238	0.029	0.34
W. Carp.	16	8	339476	3174226	0.031	-0.11
diploids	80	40	685535	3172501	0.036	-0.59
tetraploids	80	20	600938	3207613	0.034	-0.34

Table S7: Number of candidate proteins genome-wide (N candidates) and among meiosis proteins (N meiosis candidates) identified in each *A. arenosa* lineage. Last column gives a p-value of testing the enrichment of meiosis proteins among all candidate proteins (significance indicated by bold lines).

**Lineage**	**Ploidy**	**N candidates**	**N meiosis candidates**	**p-value (Fisher’s exact test)**
Pannonian	2	257	4	**1.80E-02**
Dinaric	2	211	0	1.00E+00
S. Carp.	2	91	2	5.00E-02
Baltic	2	215	3	5.19E-02
W. Carp.	2	130	0	1.00E+00
tetraploids	4	213	11	**6.51E-10**

Table S9: Hamming distances and diameters in the set of candidate meiosis proteins. Max distance 4x-2x: maximum distance from a tetraploid to a diploid haplotype, Diameter 4x: diameter of the tetraploids, Diameter 2x: diameter of the diploids.

Protein	Max distance 4x-2x	Diameter 4x	Diameter 2x
ASY1	12	2	9
ASY3	43	4	33
DYAD	15	8	11
PDS5b	36	4	28
PRD3	21	15	16
SCC4	12	3	13
SHOC1	29	6	43
SMG7	50	8	52
REC8/SYN1	24	4	22
ZYP1a	NA	NA	NA
ZYP1b	23	3	35

Table S10: Accumulation of single nucleotide polymorphisms (SNPs) after selection sweep and relative estimate of age of the selective sweeps in candidate prophase I proteins. Notes: # of SNPs: the number of variants (including synonymous and intron variation) between first and last candidate AAS, Length: the distance (in bp) between the first and last candidate AAS, SNP density = # of SNPs / Length

Protein	# of SNPs	Length (A. lyrata)	SNP density	Age
**Tetraploids**				
PRD3	133	1693	0.079	Older
REC8/SYN1	60	877	0.068	Older
ASY1	197	3713	0.054	Medium
PDS5b	303	8318	0.036	Medium
DYAD	154	11309	0.014	Younger
ASY3	20	1957	0.01	Younger
**Pannonian**				
PDS5E	62	2247	0.028	Older
ASY3	13	742	0.018	Medium
REC8/SYN1	23	2453	0.009	Younger

Table S11: Differentiation (F_{ST}, Hudson, 1992) between lineages in the subsampled dataset calculated for "neutral" synonymous and nonsynonymous SNPs genome-wide.

	Synonymous SNPs				Nonsynonymous SNPs			
	median	mean	sd	99th quantile	median	mean	sd	99th quantile
Pannonian - Dinaric	0.06	0.13	0.19	0.83	0.04	0.11	0.18	0.81
Pannonian - SCarp.	0.04	0.11	0.17	0.77	0.03	0.09	0.16	0.76
Pannonian - Baltic	0.07	0.14	0.19	0.84	0.05	0.12	0.18	0.84
Pannonian - WCarp.	0.04	0.11	0.17	0.78	0.03	0.09	0.16	0.76
Dinaric - SCarp.	0.04	0.1	0.15	0.68	0.03	0.08	0.14	0.65
Dinaric - Baltic	0.06	0.13	0.18	0.76	0.04	0.11	0.16	0.74
Dinaric - WCarp.	0.03	0.09	0.15	0.67	0.03	0.08	0.14	0.65
Baltic - SCarp.	0.03	0.08	0.13	0.6	0.01	0.07	0.12	0.58
Baltic - WCarp.	0.04	0.1	0.14	0.63	0.03	0.08	0.13	0.61
SCarp. - WCarp.	0.03	0.09	0.14	0.63	0.03	0.07	0.13	0.61
all diploids - all tetraploids	0.01	0.03	0.05	0.23	0.01	0.02	0.04	0.21

Additional files

Table S5: Amino acid substitutions (AASs) differentiated among diploid lineages which were predicted to have high functional impact.

Table S6: Amino acid substitutions (AASs) differentiated between diploids and tetraploids which were predicted to have high functional impact.

Table S8: McDonald-Kreitman test. The NAs were produced when dividing by zero in cases when no nucleotide divergences (dN) were identified.

Table S12: Orthologs of *A. lyrata* meiosis proteins in 18 plant reference genomes from the group Malvidae.

Table S13: RNASeq count tables for contrasting expression levels in the 78 meiosis genes in WCarp vs tetraploid and WCarp vs Pannonian.
